# Fabrication of High‐Density Multimodal Neural Probes Based on Heterogeneously Integrated CMOS

**DOI:** 10.1002/advs.202524260

**Published:** 2026-03-24

**Authors:** Ju Hee Mun, Miji Kim, Wooyeon Shin, Yongjun Park, Kanghwan Kim, Il‐Joo Cho, Jae Won Shim, Min Soo Kim, Sunwoo Lee, Jeongjin Kim, Changhyuk Lee

**Affiliations:** ^1^ Brain Science Institute Korea Institute of Science and Technology (KIST) Seoul Republic of Korea; ^2^ School of Electrical Engineering Korea University Seoul Republic of Korea; ^3^ Department of Electronic Engineering Hanyang University Seoul Republic of Korea; ^4^ Division of Bio‐Medical Science and Technology, KIST School University of Science and Technology Seoul Republic of Korea; ^5^ Department of Biomedical Sciences College of Medicine Korea University Seoul Republic of Korea; ^6^ KHU‐KIST Department of Converging Science and Technology Kyung Hee University Seoul Republic of Korea; ^7^ School of Electrical & Electronic Engineering Nanyang Technological University Singapore Singapore; ^8^ KIST‐SKKU Brain Research Center SKKU Institute for Convergence Sungkyunkwan University Suwon Republic of Korea

**Keywords:** bulk CMOS, MEMS process, multimodal sensor, neural probe, post‐processing

## Abstract

Neural interfaces that simultaneously capture electrical activity and cell‐type‐specific dynamics across molecularly defined populations are essential for understanding brain function. Monitoring these neural activities could help understand the complex mechanisms and disorders of the brain. While micro‐electro‐mechanical systems (MEMS) based neural probes provide multi‐sensor integration, their lack of on‐chip signal processing limits the channel density. On the other hand, complementary metal‐oxide‐semiconductor (CMOS) probes offer on‐chip amplification and multiplexing to enable high channel density, yet the high foundry tape‐out cost hinders their broader adoption. Here, we achieve a near two orders of magnitude reduction in per‐wafer cost by applying simplified post‐CMOS processing on a multi‐project wafer (MPW) while advancing the probe functionality. This approach requires only two photolithography and three etching steps for CMOS post‐processing through the decoupled sensor customization, while the front‐end circuits are defined by CMOS, multimodal sensors (electrodes and photodiodes) are defined through post‐processing. Our 13‐shank probe integrates 416 electrodes and 832 photodiodes, and complete on‐chip signal processing, enabling simultaneous high‐density electrical and optical recording. We validated the operational feasibility of the proposed system under both in vitro and in vivo conditions. Based on these results, we propose the first fully integrated active multimodal neural probe architecture for high‐density electrical and optical interfacing.

## Graphical abstract

A chiplet‐based methodology democratizes active neural probe development on standard bulk CMOS services. This yields the first probe combining high‐density electrophysiology (416 electrodes) with calcium imaging (832 photodiodes) and complete on‐chip signal processing across 13 shanks. In vivo validation demonstrates simultaneous multimodal recording from multiple brain regions, confirming practical applicability for systems neuroscience.

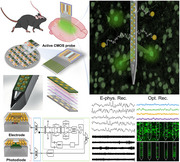

**Abbreviations**
CMOS: complementary metal oxide semiconductor
MEMS: microelectromechanical systems
MPW: multi‐project wafer
SNBR: signal‐to‐noise and background ratio
SNR: signal‐to‐noise ratio
SOI: silicon‐on‐insulator
ADC: analog‐to‐digital converter
LFP: local field potential
AP: action potential
CMP: chemical‐mechanical planarization
MOSFET: metal‐oxide‐semiconductor field‐effect transistor
SPI: serial‐to‐parallel interface
LDO: low‐dropout regulator
OTA: operational transconductance amplifier
SAR: successive approximation register
DAC: digital‐to‐analog converter
FPGA: field‐programmable gate array
FIFO: first‐in‐first‐out
IHC: immunohistochemistry
EBI: edge beading index
FIB: focused ion beam
PCB: printed circuit board
SEM: scanning electron microscopy
RIE: reactive ion etching
DRIE: deep reactive ion etching
FEOL: front‐end‐of‐line
BEOL: back‐end‐of‐line
Iba1: ionized calcium‐binding adapter molecule 1

## 1 Introduction

The mammalian brain comprises billions of neurons with extraordinary cellular diversity – recent single‐cell transcriptomic analyses have revealed over 5300 distinct cell types in the mouse brain alone [1, 2], forming exceptionally complex networks that operate across multiple spatial and temporal scales. Capturing these diverse neuronal activities requires measurement tools that can resolve single‐cell events while covering broad brain regions simultaneously. This demand has driven the evolution of neural recording technologies, from pioneering silicon‐based microelectrode arrays [3, 4] to commercially available platforms (e.g., NeuroNexus) that enabled large‐scale ensemble recordings across brain regions [5, 6].

Understanding the brain's cellular diversity demands tools capable of distinguishing molecularly defined neurons during real‐time activity. Multimodal neural interfaces – integrating electrical, optical, and stimulation modalities – have emerged as essential tools for decoding circuit dynamics. Combining optogenetic control with electrophysiological recording has enabled causal interrogation of genetically‐defined neural populations [7, 8, 9] and numerous passive and active neural probes integrating optical waveguides or light‐emitting diodes with recording electrodes have been developed for simultaneous optical stimulation and electrical recording [6, 10]. Complementarily, genetically encoded calcium indicators (GECIs) such as GCaMP have emerged as transformative tools for monitoring cell‐type‐specific neural activity by leveraging promoter‐driven expression in defined neuronal populations [11, 12, 13]. Simultaneously, electrical microelectrode arrays capture rapid action potentials and network oscillations with millisecond‐temporal resolution [6, 14]. The convergence of these modalities represents a critical frontier in systems neuroscience, correlating molecularly‐identified cellular dynamics with high‐temporal‐resolution electrophysiology across large populations [6, 14]. However, current neural interfaces struggle with a trade‐off between fabrication flexibility and scalability, limiting progress toward integrated multimodal platforms.

To overcome these limitations, two fabrication paradigms have emerged to address neural recording demands: foundry‐based passive neural probes leveraging custom silicon microelectromechanical systems (Si‐MEMS) microfabrication to explore diverse sensing architectures – such as the integration of photodiodes for optical recording [15], microfluidic channels for drug delivery [16, 17], or specialized electrode materials optimized for specific electrochemical applications [18]‐offering rapid prototyping for application‐specific designs tailored to experimental questions.

However, passive probes face fundamental scalability constraints rooted in the absence of on‐chip signal processing circuitry: amplification, multiplexing, and analog‐to‐digital conversion (ADC)–resulting in complete reliance on passive interconnect routing from each sensing element to external acquisition systems [15, 19]. This architectural limitation imposes severe restrictions: electrode density is constrained to hundreds of channels due to routing congestion; signal integrity degrades as small neural signals (50—500 µV) traverse long, high‐impedance traces susceptible to electromagnetic interference; and system complexity escalates through extensive external wiring and bulky interfaces. Despite recent MEMS innovations in geometric versatility and multimodal on‐device integration, the absence of active electronics fundamentally constrains the achievable information bandwidth and precludes scaling to the dense, large‐scale recordings necessary for decoding complex neural computations across brain regions.

Conversely, commercial CMOS foundry‐based active neural probes overcome these scalability limitations through monolithic integration of sensing elements with signal processing circuitry. Platforms such as IMEC's Neuropixels series—evolving from single‐shank (Neuropixels 1.0) [20] to quad‐shank (Neuropixels 2.0) [21] and ultra‐dense architectures (Neuropixels Ultra) [22]—demonstrate the transformative potential of on‐chip amplification, multiplexing, and digitization for achieving thousand‐channel recordings with superior signal‐to‐noise ratio and reduced system complexity [20, 21, 22, 23]. Similarly, CMOS‐compatible optical neural probes have emerged as compact and scalable alternatives to conventional head‐mounted microscopes. Approaches such as coded‐aperture imaging [24] and interference‐filter‐based imaging [25] enable on‐chip optical encoding, while the integration of angular‐sensitivity pixels has demonstrated the feasibility of 3D volumetric imaging within implantable neural probes [26, 27]. These advances highlight the potential of multimodal CMOS platforms to bridge electrical recording and optical imaging within a unified system architecture [13].

However, a critical fabrication bottleneck has emerged as limiting the broader adoption and customization of active neural probe technology. State‐of‐the‐art CMOS probes overwhelmingly rely on silicon‐on‐insulator (SOI) substrates, which enable essential post‐CMOS backend processing steps, including mechanical thinning, shaping, and release steps required for slender, implantable probe shanks [20, 21, 22]. Although the shank releasing process was simplified by leveraging the buried oxide layer as a convenient etch‐stop for backside substrate removal, SOI multi‐project wafer (MPW) services offer limited process design kit (PDK) device libraries and restricted foundry access compared to the substantially broader bulk CMOS ecosystem. Furthermore, post‐CMOS backend processing, whether SOI or bulk silicon, requires multiple photolithography, etching, and deposition steps to define probe geometry, which are increasingly difficult at chiplet scale (typically 2–5 mm) – severe edge beading effects during photoresist spin coating on millimeter‐scale dies, resulting in non‐uniform film thickness and photolithographic problems. Moreover, standard 4‐inch or 6‐inch research fabrication facilities lack tooling for reliable handling, alignment, and process control of individual chiplets during multi‐step backend sequences. The resulting barrier is substantial; while affordable MPW services provide access to advanced circuit integration, the chiplet geometries cannot be readily processed through conventional photolithography‐based backend protocols, requiring specialized processing capabilities or costly full‐wafer fabrication runs. While chiplet‐based probe enables unparalleled channel density and signal quality essential for application‐specific research, these technical and economic barriers constrain the design flexibility – custom electrode configurations, novel sensing modalities, or specialized on‐chip processing. Therefore, applications requiring simultaneous electrical and optical recording, especially for correlating molecularly defined cellular activity via GECIs with network‐level electrophysiology, remain critically underserved.

**SCHEME 1:**
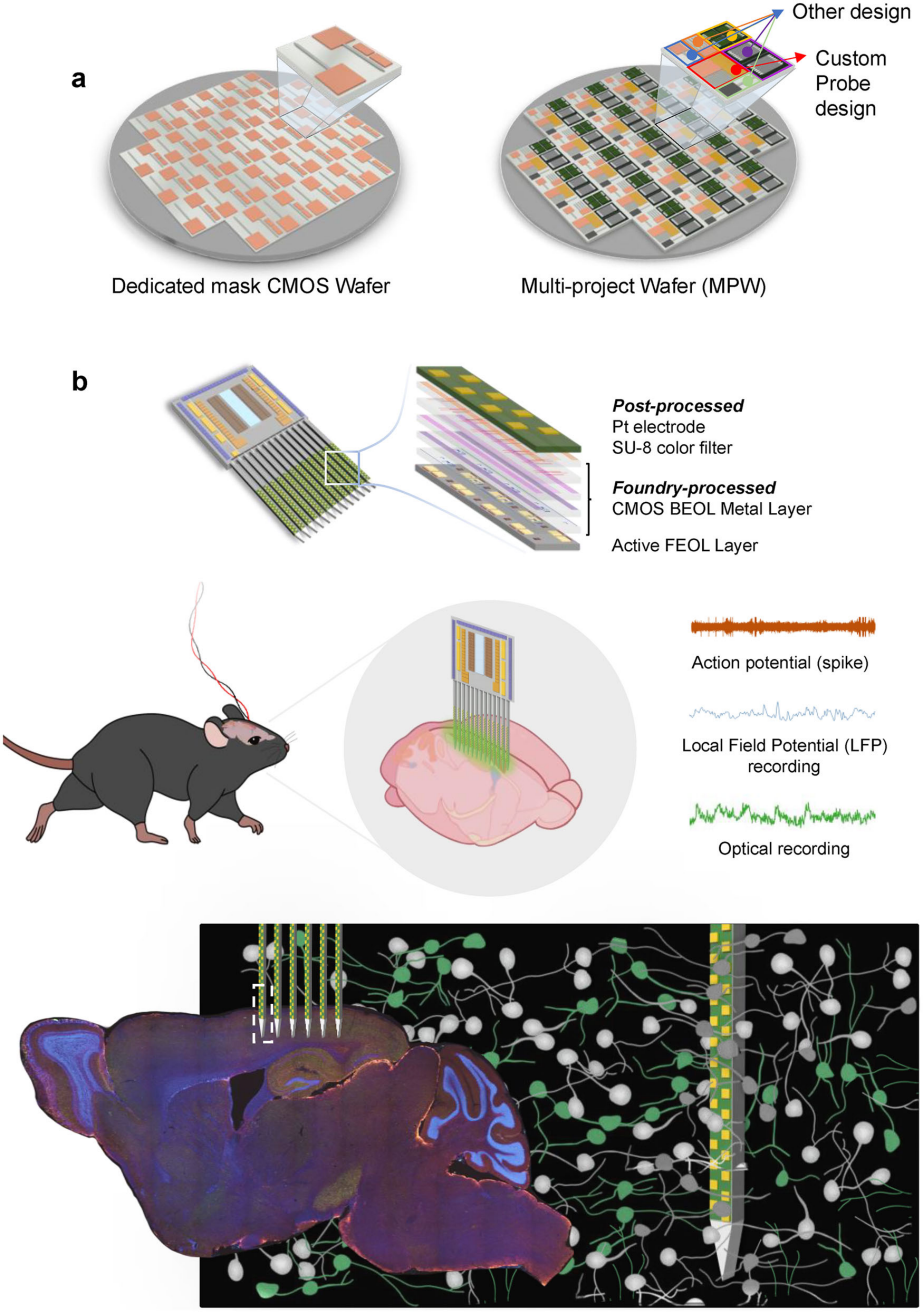
Streamlined MEMS fabrication using cost‐effective multi‐project wafer (MPW) to realize a 13‐shank multimodal CMOS neural probe (32 electrodes and 64 photodiodes per shank). a) Multi‐project Wafer (MPW) approach (right) integrates various circuit or sample designs onto one wafer by separating the mask layout, reducing the cost per design for lab‐scale research, comparing to conventional single‐project CMOS wafer using dedicated photomasks (left). b) Schematic illustration of 13‐shank multimodal CMOS neural probe and cortex implantation for recording high‐resolution neuron activities, including action potential (spike), local field potential recording (LFP), and optical recording. Our neural probe enables large‐volume coverage during insertion, allowing simultaneous recording of neuronal signals from spatially distributed regions. Its high‐density configuration of electrodes and photodiodes also facilitates multi‐region brain recording beyond localized areas.

Here, we introduce a chiplet‐based fabrication methodology that enables custom active neural probe development using standard bulk CMOS MPW services and conventional research‐scale processing tools. Our approach addresses the chiplet processing barrier through two key innovations: (1) a 3D‐printed edge‐beading suppression frame (EBSF) that enables uniform photoresist coating on millimeter‐scale dies, and (2) multi‐layer metal reinforcement structures that function as etch‐stops, eliminating SOI substrate requirements. By requiring only two photolithography and three etching steps beyond standard CMOS processing, all compatible with conventional 4‐inch research fabrication facilities, our methodology provides a pathway from affordable MPW chiplets (∼$5–10K per iteration) to functional neural probes without specialized equipment or full‐wafer commitments. (Scheme 1a) A comparative cost analysis of CMOS fabrication strategies, including MPW and full‐wafer runs, is provided in Figure S15. In addition, yield‐based estimates were incorporated to calculate the effective fabrication cost per functional probe. Table S1 further itemizes the major cost components associated with post‐processing steps to clarify the basis of the reported cost reduction.

Beyond reducing fabrication cost, this approach establishes a versatile platform for application‐specific neural interfaces and lowers the barriers to custom active probe development. It enables researchers to tailor electrode materials for optimized electrochemical sensing, design photodiodes with wavelength‐specific quantum efficiency, integrate additional sensing modalities (e.g., temperature, pH, neurotransmitters), and implement specialized on‐chip processing algorithms. In this manner, the chiplet‐based methodology combines the customizability of research‐fab passive probes with the density, signal quality, and processing integration characteristics of commercial CMOS systems.

In this study, we demonstrate a chiplet‐based fabrication methodology through the development of the first neural probe that integrates a high density of electrodes and photodiodes with complete on‐chip signal processing for both electrical and optical recording (Scheme 1b, Table S9). Our neural probe features a 13‐shank architecture (2400 µm length, 68 µm width, ∼90 µm thickness, 200 µm pitch), demonstrating unprecedented architectural complexity enabled by chiplet‐based fabrication. To our knowledge, this work represents three notable firsts in neural probe technology: (1) the first CMOS‐integrated probe enabling simultaneous high‐density electrophysiology and GECI‐based calcium imaging, (2) the first active neural probe architecture with more than eight independent shanks [28], and (3) the first system with complete on‐chip signal processing (amplification and analog‐to‐digital conversion) for both electrical and optical recording modalities. Beyond these achievements, the wider importance lies in demonstrating that chiplet‐based fabrication can deliver the customizability traditionally associated with research‐fab passive probes while maintaining channel density, signal quality, and on‐chip processing capabilities of commercial active CMOS platforms. Ultimately, this study demonstrates a lab‐accessible neural probe fabrication paradigm that democratizes custom active neural probe development for diverse neuroscience applications.

## 2 Results and Discussion

### 2.1 Multi‐Modal Neural Probe Design

**FIGURE 1:**
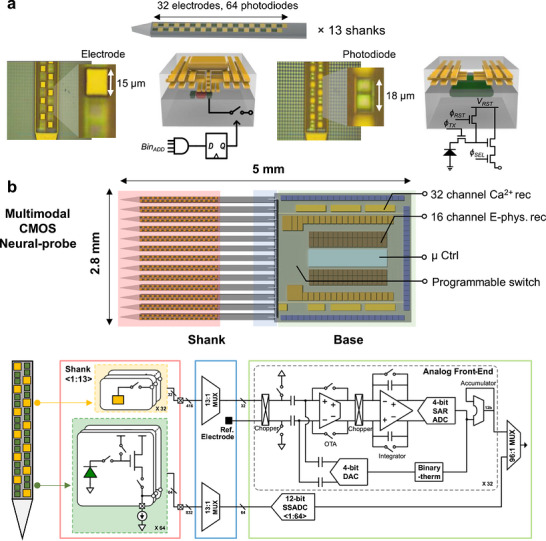
Design of components and circuit block diagram of neural probe. a) A 13‐shank multimodal neural probe was designed with 32 electrodes (15 × 15 µm/electrode) and 64 photodiodes (8 × 8 µm/photodiode) with 19 µm pitch, respectively. Electrode arrays with recording sites and switching circuits are controlled by Bin_ADD_ input (left) and photodiode arrays with pixels are integrated with readout and reset circuitry for optical signal detection (right). b) The block diagram represents a multi‐channel signal acquisition and processing system with integrated ADC architecture. This system employs multiplexers, signal amplification, noise reduction, and analog‐to‐digital conversion (ADC) to process signals from an array of pixels, integrating both single‐slope and SAR ADCs for precise digital signal output.

We developed a multimodal CMOS neural probe leveraging a chiplet‐optimized post‐processing methodology that enables sophisticated circuit integration using standard bulk CMOS technology. Unlike conventional active probes requiring wafer‐scale SOI substrates, our approach processes MPW‐scale chiplets (2–5 mm), dramatically reducing costs while enabling application‐specific customization. We fabricated a 5 mm × 2.8 mm multimodal neural probe comprising 13 shanks, each 2.4 mm in length, using a standard 180 nm bulk CMOS technology. This 13‐shank architecture, which substantially exceeds the 1–4 shanks typical of existing active probes, demonstrates the scalability of chiplet‐based fabrication and enables bilateral or multi‐region recordings over a 2.6‐mm span (13 shanks × 200 µm pitch). Each shank is equipped with 32 electrodes (15 × 15 µm/electrode) with 19 µm pitch and 64 photodiodes (8 × 8 µm/photodiode) with 19 µm pitch. We paired photodiodes with 2 µm separation to increase spatial sampling density while maintaining virtually identical detection fields (Figure 1a). This configuration achieves 64 photodiodes per shank, providing spatial resolution suitable for single‐cell level calcium imaging. The paired‐photodiode architecture also enables future multi‐color filter integration for simultaneous imaging of multiple fluorescent indicators [29], or as recently demonstrated for tracking neural populations across weeks using static markers alongside dynamic calcium signals [30]. With 416 electrodes and 832 photo‐pixels, this architecture would require at least 1248 individual wire connections in a conventional passive neural probe – a configuration that becomes mechanically infeasible beyond >100 channels due to routing congestion and bond pad constraints [31, 32]. This fundamentally illustrates why on‐chip amplification, multiplexing, and digitization enabled by commercial CMOS foundries are essential for achieving single‐cell‐resolution multimodal recording across large tissue volumes. Each electrode and photodiode is connected to the metal interconnects through a via with 16 wires located on the left and right sides of each shank. This space‐efficient routing is enabled by the commercial foundry process, which provides chemical‐mechanical planarization (CMP) for multiple metal layers and MOSFET switching capabilities that are unavailable in typical research fabrication facilities, further demonstrating the advantages of our standard CMOS‐based approach.

A further innovation in this approach is the multi‐layer metal reinforcement structure surrounding each shank. We utilized all six metal layers (M1 through M6) in the TSMC 180 nm process, creating a continuous 5 µm wide protective wall of M6‐to‐M1 layer around each shank perimeter (Figure S1, Supporting Information). This structure serves multiple functions: (1) mechanical reinforcement similar to MEMS cantilever designs [33, 34], (2) electrical connection to the reference electrode at the shank tip, and (3) etch‐stop protection for underlying circuit layers. The 4 µm thick M6 layer at the shank boundary effectively prevents damage to lower metal layers during aggressive etching processes, with aluminum serving as an etch stop for both oxide and silicon etching chemistries. This metal‐reinforced design, combined with the edge‐beading suppression methodologies detailed in Section 2.3, enables robust chiplet‐scale fabrication that would otherwise fail due to non‐uniform photoresist coating and etch non‐uniformities at small die edges.

### 2.2 On‐Chip Multi‐Modal Neural Signal Readout Circuits

The multimodal sensing capabilities described above depend critically on integrated signal processing circuits that must simultaneously handle low‐amplitude electrical signals (≈50–500 µV action potentials) and optical signals spanning three orders of magnitude in intensity (1–100 nWmm^−2^). This dual‐modality requirement, supported by commercial foundry‐provided transistors, capacitors, and analog‐to‐digital converters, exemplifies the circuit complexity achievable only through CMOS integration.

The proposed neural probe incorporates high‐density, low power analog and mixed signal CMOS circuitry for concurrent measurement of optical and electrical neural signals (Figure 1b). Each photo‐pixel, which converts optical signals (photons) to photocurrent, comprises a low‐noise, rounded‐corner N‐well photodiode and three transistors (3T) pixel: one for reset, one for source‐follower voltage buffering, and one for selectively connecting pixels to time multiplexed single‐slope analog‐to‐digital conversion (SSADC) circuit integrated in probe base. The arrangement, conventional in CMOS process, enables a low‐transistor‐count integrating amplifier with <25 pW noise effective power readout for many pixels. Furthermore, the readout noise is reduced by implementing correlated double sampling. The in‐shank circuit for electrophysiological (e‐phys.) signal amplification and processing is minimized to avoid photoelectrical artifact [35] in the range of 30 𝜇V–1 mV depending on fluorescence excitation optical power, effective optical coupling coefficient (α), and electrode material [17]. Amplifying and A/D converting the e‐phys signal heavily in the base of shank requires an efficient signal routing, therefore, we implemented a hierarchical multiplexing scheme. At the shank level, the 32 electrodes are organized into four selectable groups (8 electrodes each). These groups interface with an analog switch matrix (multiplexer) at the shank‐to‐base connection, programmable via a serial‐to‐parallel interface (SPI). The architecture enables users to select all four groups from a single shank or distribute them across multiple shanks (e.g., group A from Shank 1, groups B and C from Shank 3, and group D from Shank 13), thereby allowing targeted recording from specific brain regions. The electrical recording circuitry incorporates 32 mixed‐signal front‐end circuits (12‐bit, 8 kS/s), each equipped with a low‐dropout regulator (LDO) to mitigate power coupling. These front‐end circuits accommodate ± 32 mV voltage spans to manage electrode offsets, neural signals, and stimulation artifacts. Embedding the ADC within a capacitive feedback loop to optimize the input range, employing chopper‐stabilization, and using a current‐input loop filter are utilized to decrease low‐frequency noise and offset, which enables a fast return to baseline [36]. The 4‐bit 250×‐oversampled successive approximation register (SAR) ADC yields 12‐bit resolution using a charge‐redistribution digital‐to‐analog converter (DAC). This design maintains stable gain irrespective of temperature and process variation. Additional features include offset storage during reset periods and out‐of‐band noise conversion, which support small capacitance and high input impedance. The entire readout system can be software‐defined by an external FPGA connecting via an SPI programming interface, which enables real‐time programming and monitoring. Continuous recording is facilitated by an on‐chip output data serializer and a synchronized FPGA with a first‐in‐first‐out (FIFO) digital buffer architecture. This signal processing pipeline, extending from electrode/photodiode to digitized output, illustrates the capabilities of analog and digital mixed‐signal computational function integration enabled by commercial CMOS processes, surpassing the achievements typically attainable with research fabrication methods.

### 2.3 CMOS Post‐Processing Fabrication Optimization

We employ AZ 10XT Photoresist (520 cP) (Merck Performance Materials GmbH) in our post‐CMOS fabrication on standard bulk CMOS chiplets. The photoresist provides sufficient thickness (∼14 µm) and stability required for prolonged etching concomitant with CMOS post processing, hence eliminating the need for specialized substrates (e.g., silicon‐on‐insulator), metal hard‐mask [37, 38] or niche equipment [39, 40]. With a photoresist selectivity of 40, a single coating of the photoresist can withstand front‐side back‐end‐of‐line (BEOL) dielectric removal (∼13.2 µm) and front‐side silicon (Si) deep reactive ion etching (DRIE) for shank definition, and a single back‐side coating can withstand the DRIE for thinning and release across diverse MPW specifications (details in Supplementary Note S1).

**FIGURE 2:**
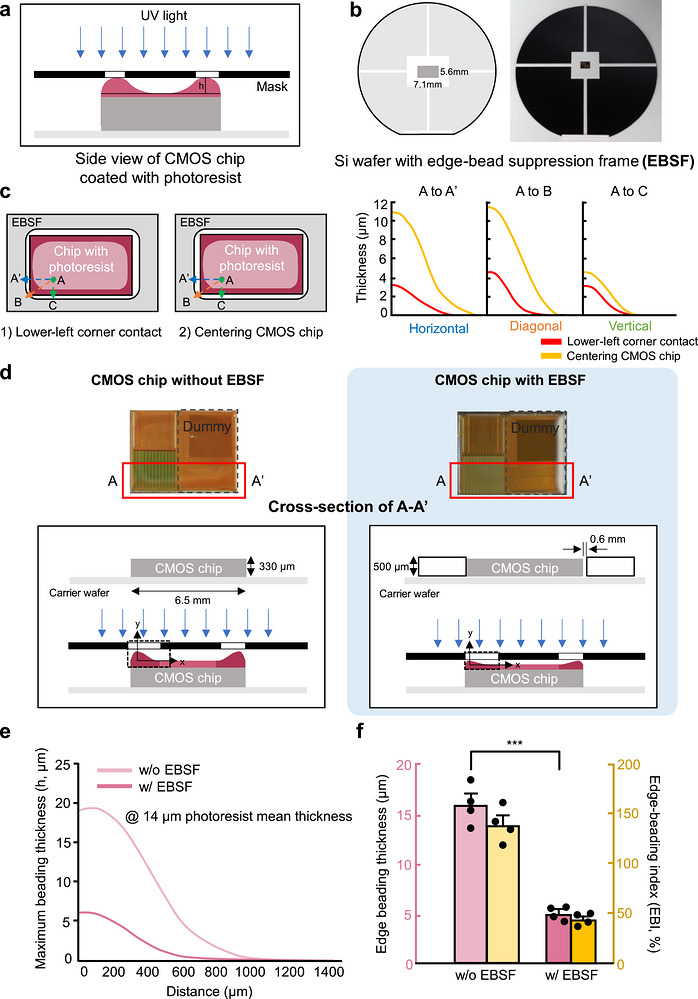
Minimizing edge‐beading formation via 3D‐printed edge‐beading suppression frame (EBSF). a) Photolithography‐related problems such as focus deviation, light scattering, and low pattern fidelity due to edge‐beading formation. b) Edge‐beading suppression frame (EBSF) was designed with 3D printer, which includes 7.1 mm × 5.6 mm hole at the center to accommodate the CMOS chip, and placed on the Si wafer. c) Optimization of minimum edge‐beading thickness using EBSF. Two placement configurations were evaluated; 1) positioning the chiplet with lower‐left corner contact and 2) centering the chiplet within the EBSF. Corner‐contact alignment minimizes the gap at the probe region (left: 96 ± 3 µm, bottom: 57 ± 6 µm), while center alignment provides uniform clearance on all sides (∼300 µm nominal). Measurements of edge‐beading thickness to horizontal (A to A’), diagonal (A to B), and vertical direction (A to C) from the planarized center show that lower‐left corner contact (red plot) suppresses edge‐beading formation more effectively than the centering placement (yellow plot). d) CMOS chip without EBSF (left) exhibits a broad, relatively thick photoresist along the edge (A‐A’), whereas CMOS chip with lower‐left contact EBSF (right) shows a considerable reduction in photoresist thickness at the edge (A‐A’) under the identical recipe. e) Maximum photoresist thickness at the edge of CMOS chip with and without EBSF was 6.0 and 19.4 µm, respectively. f) With modified recipe and EBSF (target photoresist: ∼14 µm), the edge‐beading index (EBI) was 3.2 times lower than edge‐beading index without EBSF (w/o EBSF EBI = 162%; w/ EBSF EBI = 50%).

Despite its benefits, such high photoresist thickness often leads to edge‐beading (accumulation of photoresist at chip edges due to surface tension [41], negligible on full wafers but dominant on millimeter‐scale chiplets [42]) which scales with photoresist viscosity and thickness. Figure 2a illustrates the issue with a quantified metric, edge‐beading index (EBI = (h_edge_ − h_center_)/h_center_ × 100%, see Supplementary Note S2). As many mitigation techniques such as spray coating [43], are unreliable and/or inaccessible [41], we have 3D‐printed (M160, Moment) a polylactic acid edge‐beading suppression frame (EBSF) with optimized height and controlled gap spacing. This EBSF was mechanically attached to the carrier wafer surrounding the chiplet to ensure uniform centrifugal loading and consistent thickness distribution (Figure 2b). The elevated frame boundary reduces surface‐tension‐driven meniscus formation at chiplet edges by providing a nearby vertical surface that preferentially wets the photoresist [44, 45]. The gap between frame and chiplet was designed to accommodate the dimensional variability of 3D‐printed frames (315–411 µm diagonal inward deviation) while maintaining effective edge‐bead suppression. For asymmetric probe layouts, corner‐contact alignment minimizes the gap at the functional region (Figure 2c–f; Figures S3 and S4). The combination of parameter optimization and the EBSF reduced the edge‐to‐center thickness variation from 19.4 to 6.0 µm (EBI from 162% to 50%, a 3.2‐fold improvement), an acceptable tolerance for contact lithography [46], and this variation reduction was reproducible across all 13 shanks of the probe array (Figures S3 and S4; details in Supplementary Notes S3 and S 4).

**FIGURE 3:**
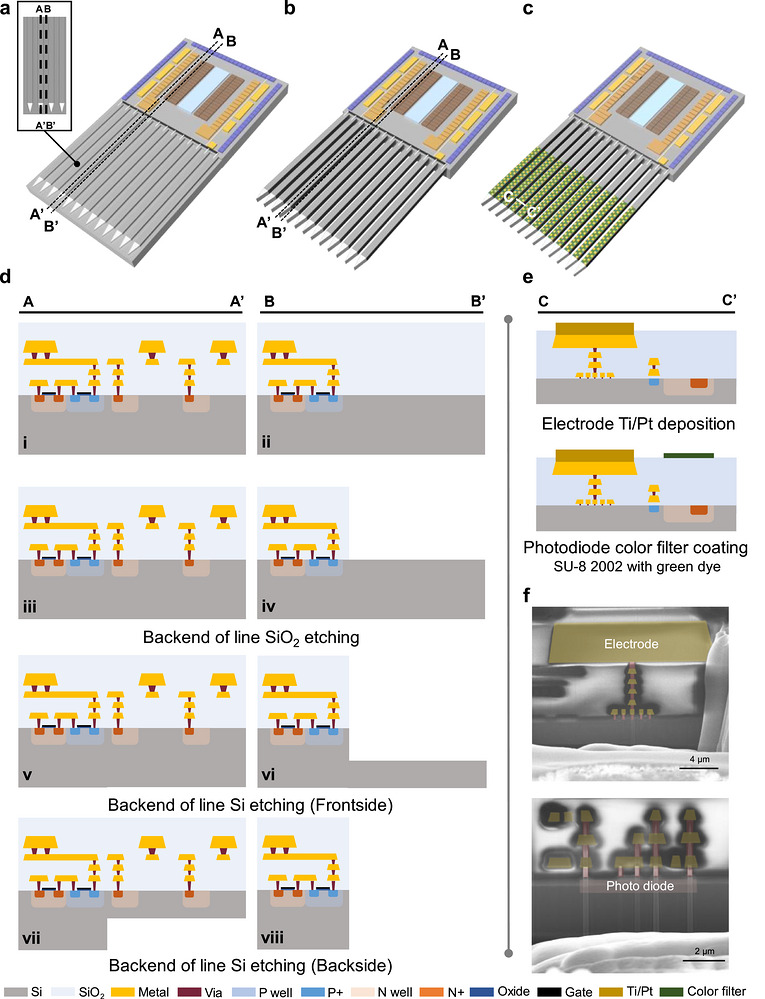
Schematic illustration of the overall MEMS process with cross‐section structures for defining the probe shank. After the FEOL process, the multi‐modal 13‐shank neural probe with 32 electrodes and 64 photodiodes per shank was manufactured. a–c) Through RIE and DRIE processes to remove SiO_2_ and Si, we obtained shank released structure as illustrated. d) Cross sections of base‐to‐shank (A‐A’) and base‐to‐inter‐shank regions (B‐B’) were demonstrated after each etching process. e) After releasing 13 shanks, Pt deposition was performed on electrodes, and green color filter array was coated on photodiodes with photolithography and the cross sections of the functionalized part of shank after each procedure were mentioned (C‐C’). f) Through FIB, we confirmed that the structure of electrodes and photodiodes matches well with our designed layer.

Figure 3 illustrates the subsequent etching processes. After removing the CMOS inter‐metal‐dielectric (IMD) layer, which is largely Si_X_O_Y_N_Z_, between the shanks (Figure 3a,d), the front‐side DRIE removes silicon to define individual probe shanks and the back‐side DRIE removes bulk silicon (Table S3) to achieve final shank thickness and the release of the shanks (Figure 3b–d). The final shank thickness is ∼90 µm, just above the mechanical fragility threshold.

This chiplet‐based methodology, requiring only two photolithography and three etching steps enabled by EBSF, demonstrates that application‐specific active neural probes can be developed using standard MPW services and conventional research‐scale tools, bridging commercial CMOS integration with custom sensor fabrication.

### 2.4 Pt Deposition and Integration of Color Filters on Photodiodes for Electrical and Optical Analysis

While aluminum is the standard metallization material in CMOS back‐end‐of‐line (BEOL) processing—used in legacy processes (≥ 110 nm) as Al or Al‐Cu alloys, and replaced by Cu in advanced nodes (≤ 90 nm)—it presents significant challenges when used directly as neural recording electrodes due to high impedance. Extensive comparative studies exist in the literature examining electrode materials optimized for low‐impedance neural recording applications [47, 48]. To address these limitations, we implement platinum coating on electrode sites using our established protocol (Figure 3e). The input impedance of our AFE is sufficiently high (≈ 28 MΩ at 1 kHz), enabling the use of planar Pt electrodes that provide adequate impedance while maintaining a simplified fabrication process. The platinum (Pt) electrodes were deposited with a titanium (Ti) adhesion layer using electron‐beam (E‐beam) evaporation, followed by a lift‐off. To ensure complete and reliable coverage, the electrode patterns were defined as 17 × 17 µm, providing a 1 µm margin relative to the nominal electrode dimensions. Electrodes of comparable dimensions have reported similar impedance levels in prior studies [28, 49] and our measured results show comparable performance, as characterized in Figure S10.

The subsequent validation focuses on the multi‐shank CMOS integration, color filter performance, and complete system‐level characterization (Sections 2.5–2.9).

To effectively acquire fluorescence signals from various types of neurons, suppression of the excitation light is crucial due to its significantly higher intensity compared to the emission light. We integrated absorptive color filters directly on the photodiode to achieve strong excitation rejection (Figure 3e) [15, 50, 51], achieving strong rejection of excitation while maintaining >30% transmission at emission wavelengths. The detailed fabrication process of the color filters is described in Section 4.4.

Integrating a color filter with active CMOS on chiplets presents unique challenges: (1) alignment accuracy at sub‐µm scales across small die edges, (2) thermal budget constraints that limit post‐processing to <150 °C to preserve CMOS circuits, and (3) photolithography on non‐planar surfaces after shank release. These constraints preclude conventional filter integration methods and necessitate the low‐temperature, chiplet‐compatible approach. To compensate for potential misalignment during photolithography, color filter patterns were intentionally designed with 3 µm oversizing relative to the photodiode array, ensuring complete coverage despite alignment tolerances of standard contact mask aligners. The measured alignment accuracy of 1.20 ± 0.03 µm confirms robust process control within equipment capabilities. The complete fabrication process remains compatible with low‐temperature (<150 °C) BEOL processing, enabling integration without degrading CMOS circuit performance [52, 53, 54]. This fabrication approach demonstrates that sophisticated multimodal functionality can be achieved through accessible, cost‐effective post‐CMOS processing.

### 2.5 Microstructural Design and Validation of Fabricated Neural Probe

We successfully fabricated a 13‐shank CMOS‐integrated neural probe array featuring co‐located optical and electrical recording modalities on a single monolithic substrate (Figure 3c). The fabricated device structure was characterized by focused ion beam (FIB) milling to obtain high‐resolution cross‐sectional images of the electrode‐photodiode interface (Figure 3f) enabling precise verification of layer integrity and alignment within the multilayered probe architecture. The completed device was wire‐bonded to a custom‐designed PCB to enable operation of 832 photodiodes and 416 electrodes, ensuring robust electrical interfacing for subsequent characterization.

**FIGURE 4:**
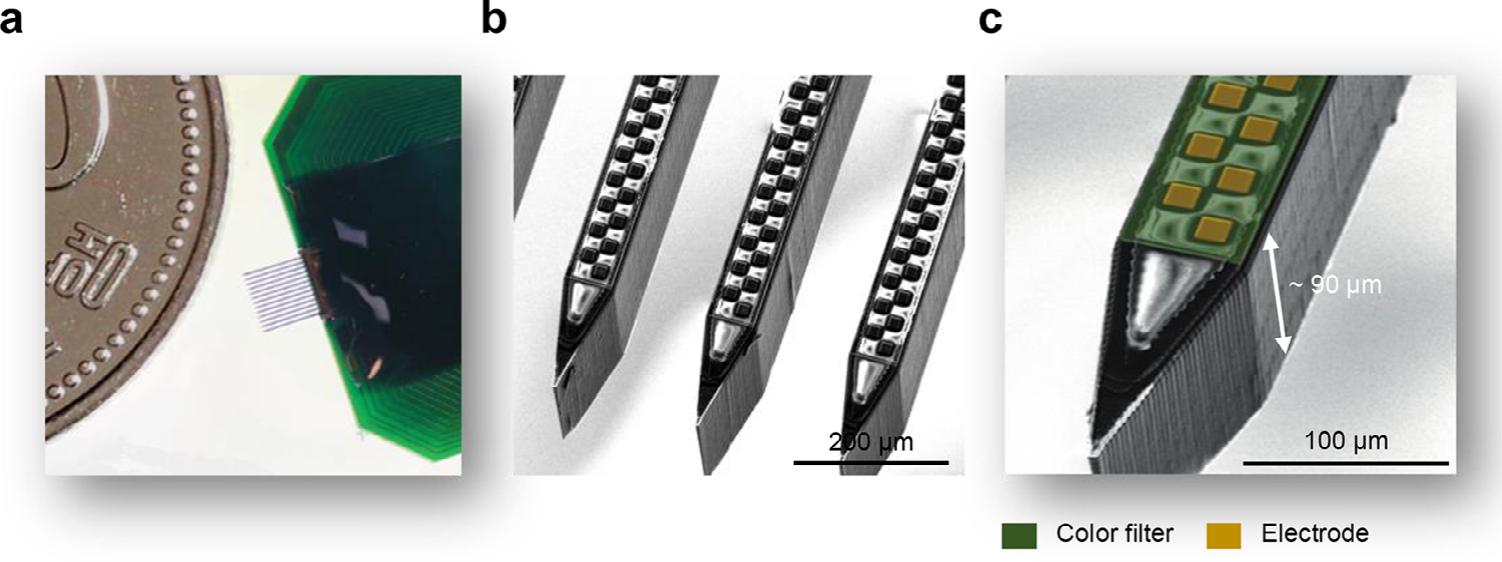
Scanning electron microscopy (SEM) images of multi‐modal neural probe. a) A coin‐scale visual confirmation was conducted to ascertain that the shank size of the neural probe was sufficient for insertion into the brain. b, c) The thickness of the shank was measured as 89.78 ± 1.61 µm, which was characterized through SEM analysis. Pt electrodes and color filter are highlighted in false‐color for clarity.

Scanning electron microscopy (SEM) analysis confirmed the structural integrity of all 13 shanks (Figure 4a–c; Figure S9, Supporting Information). The combined BEOL and Si front‐etch depths establish the upper limit of shank thickness, with final backside etching achieving shank thicknesses of 89.78 ± 1.61 µm (n = 26 shanks across 2 chips). Statistical analysis revealed shank width uniformity of 70.03 ± 0.66 µm, demonstrating the reproducibility of the chiplet‐based post‐processing methodology. The SEM images, presented in false color for enhanced contrast, clearly delineated the probe's shank geometry, electrode layout, and color filter integration (Figure 4b,c), confirming the successful formation of the multilayer architecture and preservation of critical features required for neural recording and stimulation.

### 2.6 Evaluation of Color Filter Uniformity and Optical Performance

To evaluate the optical response under conditions relevant to neural fluorescence imaging, wavelength bands corresponding to the excitation and emission spectra of GCaMP were selected as representative targets. These spectral references enable benchmarking of the photodiodes and color filter performance, providing a basis for future in vivo calcium imaging applications. GCaMP exhibits green fluorescence with peak excitation at 470–488 nm and emission at 510–525 nm when bound to Ca^2^^+^ [12, 55, 56]. To evaluate color filter performance, we selected 480 and 520 nm as representative excitation and emission wavelengths, respectively, which align with the practical emission bandwidth of GCaMP indicator [11, 12].

**FIGURE 5:**
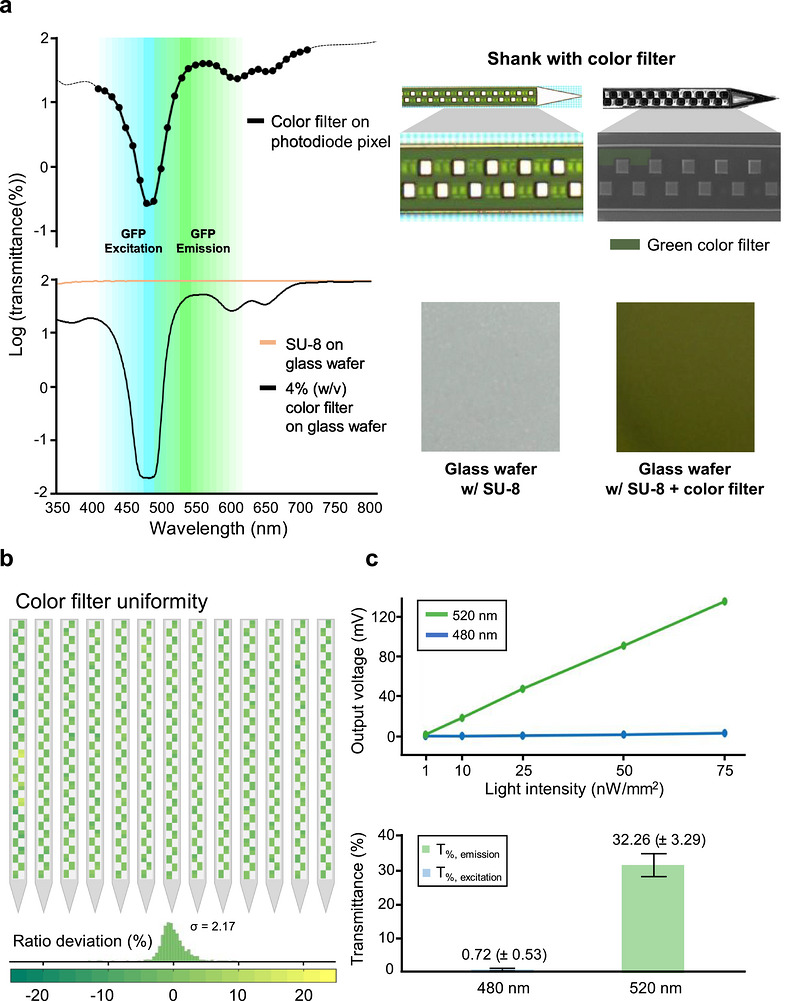
Comprehensive optical characterization of the on‐chip color filter. a) Spectral transmittance of the integrated color filter measured via photo‐pixel response (top) and validated with VIS‐NIR spectrophotometry (Cary 5000, Agilent) on control samples (bottom) fabricated on glass wafers (left: glass wafer w/ SU‐8, right: glass wafer w/ SU‐8 + color filter). b) Color filter uniformity map of the relative rejection ratio deviation (R_reject_ = V_520_/V_480_, where pixel voltage outputs under 520 and 480 nm illumination at 5 nWmm^−2^) across 832 photo‐pixels distributed over 13 shanks. The majority of pixels exhibited deviations within ± 2.2% of the mean rejection ratio. c) Photo‐pixel responses to 480 nm (blue line) and 520 nm (green line) illumination at optical powers ranging from 1 to 75 nWmm^−2^ (n = 5 trials per power) (top). Repeated measurements yielded mean transmittances of 32.26 ± 3.29% at 520 nm and 0.72 ± 0.53% at 480 nm (bottom).

Figure 5a shows the spectral characteristics of the integrated color filter. Monochromatic illumination (5 nWmm^−2^) was applied to the photo‐pixels from 400 to 700 nm in 10 nm increments, and transmittance was calculated as the ratio of filtered pixel voltage output to an unfiltered reference pixel on the same shank, measured under identical illumination conditions. Within the excitation band (470–490 nm), the measured transmittance ranged from 0.27% to 0.94% and 12.7–33.7% at the emission wavelength range (510–530 nm), achieving sufficient discrimination of GCaMP fluorescence changes from excitation light crosstalk. To validate the photo‐pixel‐based spectral characterization, we fabricated control samples on glass wafers with SU‐8 and SU‐8 with color filter under the identical process recipe applied to the neural probe shanks. Optical transmittance spectra were acquired using a VIS–NIR spectrophotometer (Cary 5000, Agilent) at normal incidence, which exhibited an average transmission of 27.3% at the emission band and <1% at the excitation band. The transmittance of the on‐pixel color filter was consistent with the glass wafer control measurement, demonstrating measurement fidelity. Close agreement between electrical (photodiode) and optical (spectrometer) measurements indicates that the integrated CMOS sensor accurately reports the filter's spectral characteristics. Figure 5b quantifies color filter uniformity across the entire probe array by plotting the deviation of rejection ratio, defined as R = V_520_/V_480_, where V_520_ and V_480_ represents pixel voltage outputs under 520 and 480 nm illumination (5 nWmm^−2^), respectively. The relative deviation from the mean rejection ratio visualized across 13 shanks reveals slightly elevated variation (14% from the mean) in pixels near the perimeter of Shank 1, likely attributable to edge effects during spin‐coating. The majority of pixels exhibited deviation within ± 2.17% of the mean, demonstrating the robustness of the fabrication process for large‐area integration. To assess the color filter's performance under varying illumination intensities, we measured pixel output voltage as a function of incident optical power from 1 to 75 nWmm^−2^. As shown in Figure 5c, the pixel response to 520 nm emission exhibited strong linearity (R^2^ = 0.9997, n = 5 trials) across the entire dynamic range. In contrast, the response to 480 nm excitation remained near the noise floor across all intensities, confirming robust excitation rejection. To quantify measurement repeatability and noise contributions, transmittance measurements were repeated five times at each optical power level. Within the measured intensity range, the transmittance averaged 0.72 ± 0.53% at 480 nm, where the low signal level approaches the photodiode's dark current noise floor, resulting in quantization‐limited precision due to the 12‐bit ADC resolution. The transmittance averaged 32.26 ± 3.29% at 520 nm, in which photon shot noise became dominant due to an approximately 45‐fold increase in photon flux.

### 2.7 In Vitro Validation of the Multimodal Sensing Interface

**FIGURE 6:**
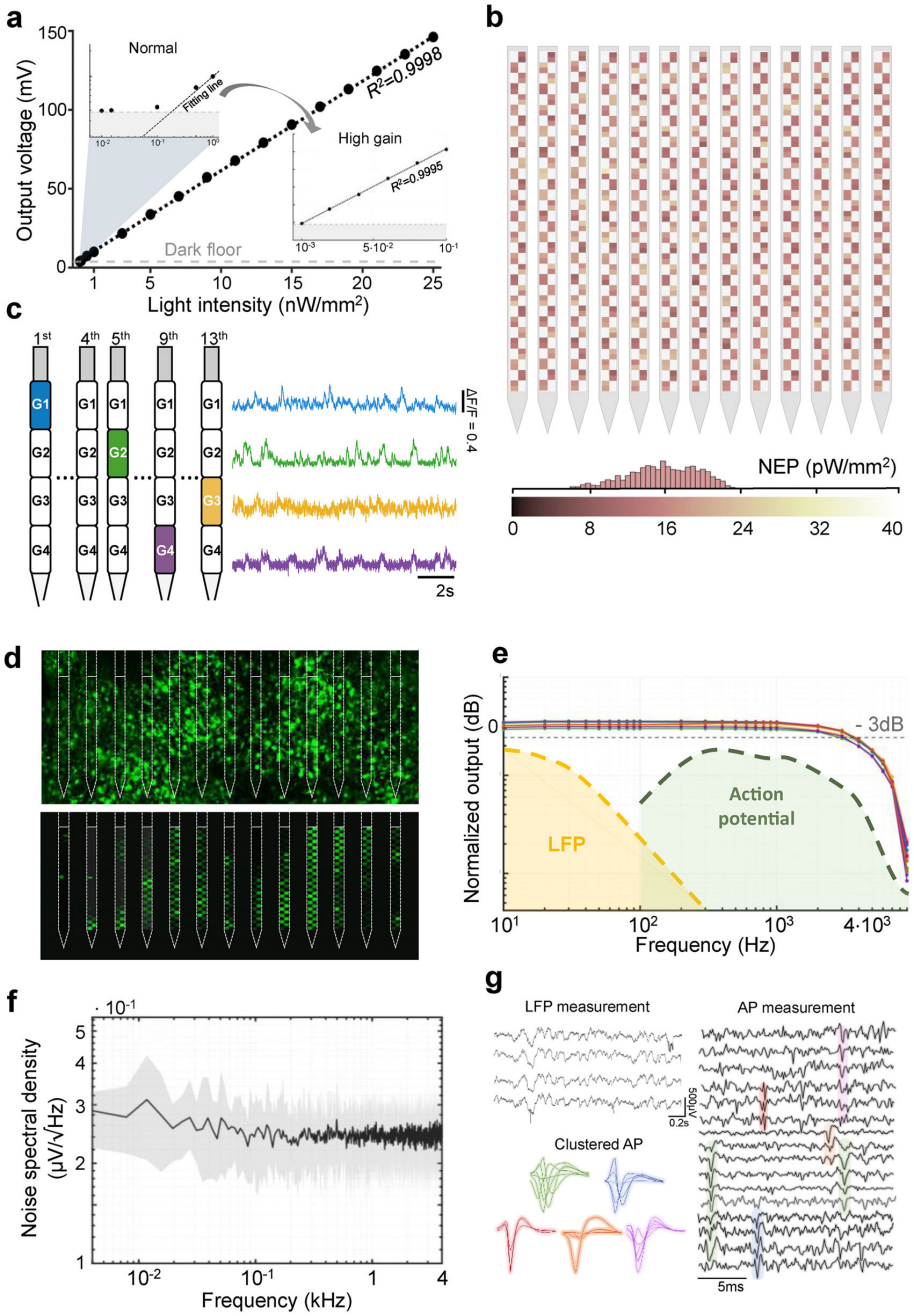
Optical and electrical recording circuit characterization of the multimodal CMOS neural probe. a) Photo‐pixel output voltage as a function of incident 520 nm irradiance measured in normal mode (400 fps) exhibiting linearity (R^2^ = 0.9998) from 1 to 25 nWmm^−2^. The inset shows operation in high‐gain mode (25 fps) extending linearity down to 1 nWmm^−2^ (R^2^ = 0.9995). b) Noise‐equivalent power (NEP) derived from the dark RMS noise across 13 shanks. The corresponding SNR at 1 nWmm^−2^ is 62. c) Time series GCaMP6 recording from four regions of interest. d) Image of GCaMP6 fluorescence projected onto 13 shanks through relay optics. e) Averaged frequency response of 16 recording electrodes (four per shanks 1, 5, 9, and 13) under sinusoidal test signals (10 Hz‐9 kHz, 10 mV_PP_), showing a −3 dB cutoff at 3.75 kHz. f) Readout noise spectral density of 16 electrodes, plotted as the mean (solid line) with standard deviation (shaded ribbon). g) Recording data of pre‐recorded neural signals applied to the electrodes, showing simultaneous acquisition of low‐, high‐frequency signals. AP clustering was performed using Kilosort 4.0, isolating five units.

Photo‐pixel performance was characterized by measuring the output voltage as a function of incident irradiance at 520 nm. In the normal imaging mode (400 fps), the sensor exhibited strong linearity (R^2^ = 0.9998) over an irradiance range of 1–25 nWmm^−2^ (Figure 6a), confirming a proportional relationship between incident irradiance and output voltage. At low irradiance levels, the response in this mode approached the noise floor and became noise‐limited. In this measurement, the normal imaging mode employed a frame structure in which a fixed fraction (10%) of each frame period was allocated to reset and settling, after which the photo‐generated current was integrated over the remaining frame duration. As a result, the integration period at 400 fps constrains the accumulated signal at low irradiance levels, limiting the achievable output amplitude. Consistent with this configuration, operating at a high‐gain mode (25 fps) extends the integration time under the same readout scheme, thereby increasing the effective optical gain and restoring linearity down to 1 nWmm^−2^ (Figure 6a inset), at the expense of temporal resolution. The frame rate determines the integration time of the photo‐pixel, which in turn influences the relative weighting of different noise mechanisms in the optical readout. Prior analyses of CMOS image sensors indicate that short integration times accentuate readout‐related noise sources such as reset operation noise, whereas longer integration times allow photo‐current‐related shot noise and flicker noise to contribute more significantly at the integration node [57, 58].

In Figure S11a, the impact of photo‐pixel operation on the electrical recording channels is evaluated under two illumination conditions (60 pWmm^−2^ and 1 nWmm^−2^). At 25 fps, no distinct photoelectrical artifact is observed even under increased illumination (below 1%). At 400 fps, a modest RMS noise increase relative to the baseline is observed, which is attributed to weak capacitive coupling of high‐frequency photo‐current–induced charge through parasitic paths, as illustrated by the layout diagram in Figure S11b, rather than to instability or direct electrical interference. Figure 6b shows the Noise‐Equivalent Power (NEP) derived from the dark RMS noise across 13 shanks. At the lower limit of physiological relevance (1 nWmm^−2^), this corresponds to SNR = 62, which is sufficient for resolving GCaMP calcium transients. To validate spatial imaging performance under controlled conditions, we projected GCaMP6 calcium emission onto the probe through relay optics, using ex vivo hippocampal tissue as an optical phantom. This approach provides ground truth comparison: the relay optics creates a mirror image of biological fluorescence dynamics on the chip surface, enabling quantitative assessment of spatial resolution and temporal fidelity without confounding variables from live animal experiments. Time series recording from four regions of interest (ROI, Figure 6c) successfully resolved single‐cell level transients (ΔF/F_0_ = 15–40%) and discriminated temporal patterns, confirming detection capability for physiological calcium dynamic recording. Figure 6d demonstrates preserved optical detection capability across all 13 shanks (832 photodiode pixels total).

Electrical performance was characterized using 16 electrodes (4 per shank from Shanks 1, 5, 9, 13). To characterize the frequency response of the recording circuit, sinusoidal test signals (10 Hz–9 kHz, 10 mV_PP_) were applied via a waveform generator to electrodes. The recording front‐end employs a chopper‐stabilized operational transconductance amplifier (OTA)‐based integrator followed by a 4‐bit oversampled SAR ADC (250× oversampling, 12‐bit effective resolution) enclosed within a capacitive feedback loop. A charge‐redistribution DAC provides feedback at the integrator input, and the effective closed‐loop gain is determined by the capacitor ratio C_AC_/(C_DAC·_V_DAC,fs_) independent of process and temperature variations. Under a nominal gain of 23 dB, the system exhibited a −3 dB cutoff frequency of 3.7 kHz (Figure 6e), which covers the bandwidth of extracellular neural signals reported in prior work [59, 60]. Figure 6f shows the readout noise spectral density, plotted as a solid line with the deviation across electrodes shown as a shaded ribbon. Integration of the noise power over the LFP (1‐300 Hz) and AP (300‐3.7 kHz) bands yielded RMS noise levels of 5.83 ± 2.1 and 10.8 ± 0.7 µV, respectively, supporting reliable neural activity recordings. Electrical performance was validated using pre‐recorded neural signals obtained from CA1–MEC recordings (LFP: 0.1‐300 Hz, 100–500 µV; AP: 300 Hz–5 kHz, 50–150 µV), which were applied to the electrode. This controlled validation approach enables (1) reproducible characterization across all 416 electrodes without biological variability, (2) direct comparison against established neural recording benchmarks, and (3) systematic assessment of signal chain integrity from electrode interface through digital output. Figure 6g demonstrates simultaneous capture of LFP oscillations (1‐10 Hz) and action potential (AP) waveforms, with spike sorting (Kilosort 4.0) [61] successfully isolating five units.

To assess the biological safety of the system, we evaluated device‐induced temperature rise under operating conditions. The temperature increase at the shanks remained below 0.2 °C (Figure S12a), well under the commonly accepted safety threshold (∼1 °C) for implanted neural devices [62, 63]. A temperature increase (1.4 °C) was observed only around the power supply routing of the headstage region, outside brain tissue.

### 2.8 In Vivo Validation of Multimodal Neural Probe Recording

**FIGURE 7:**
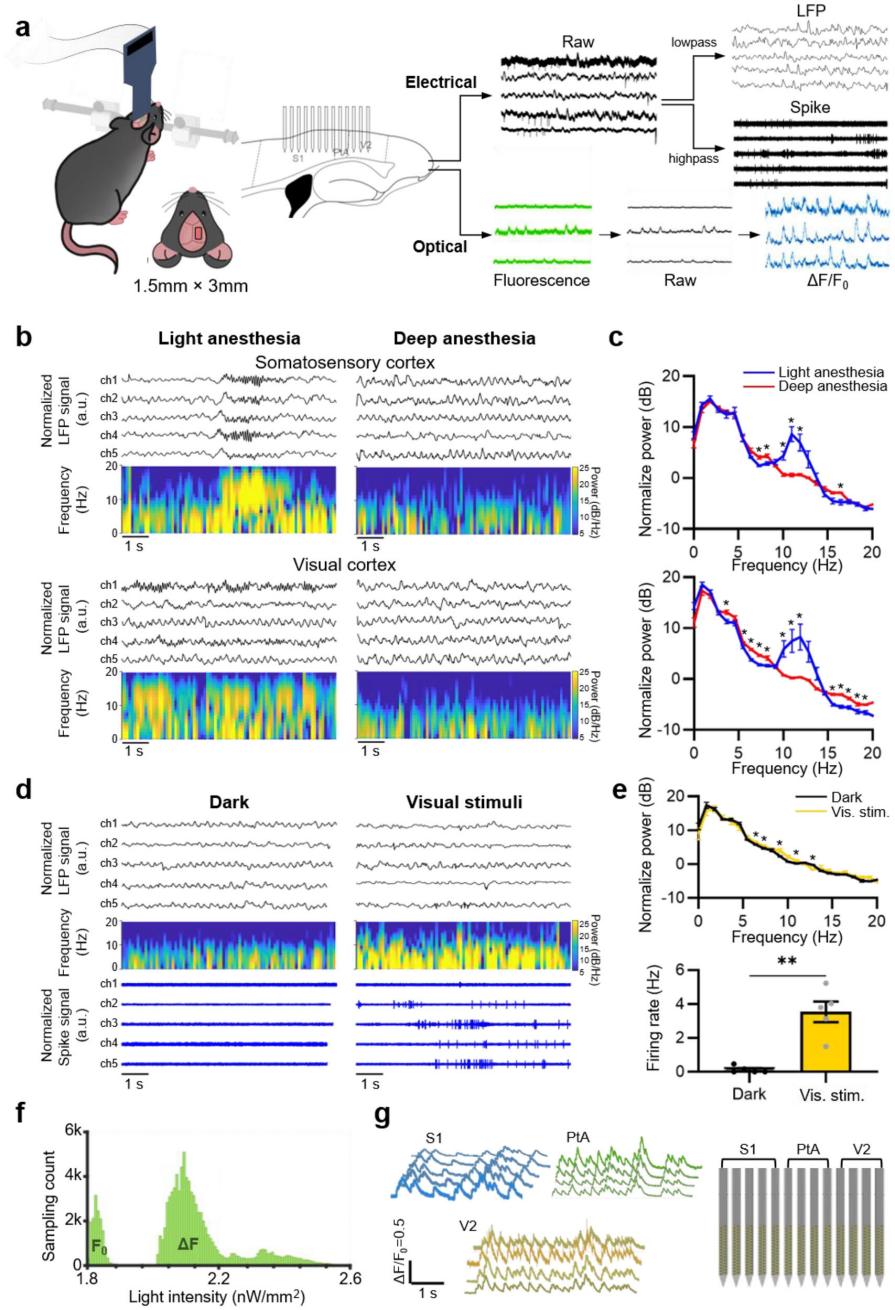
In vivo electrical and optical recordings of the multimodal CMOS neural probe. a) Schematic illustration of the overall in vivo multimodal recordings and analysis process. b) Comparison of LFP signals between light anesthesia and deep anesthesia states in the somatosensory cortex and visual cortex. c) Power spectral density (PSD) comparison between light and deep anesthesia conditions. Data are measured with mean with standard error of the mean (S.E.M.) with paired t‐test at each frequency (N = 5 channels, *p < 0.05). d) Comparison of LFP and spike signals between dark conditions and visually evoked conditions (lighted) in the visual cortex. e) Power spectral density (PSD) comparison between dark and lighted conditions. Data are measured with mean with standard error of the mean (S.E.M.) with paired t‐test at each frequency (N = 5 channels, *p < 0.05). The average firing rate was also compared between conditions. Data are measured with mean with standard error of the mean (S.E.M.) with paired t‐test (N = 5 channels, **p < 0.01). f) Histogram of effective incident light intensity at the photo‐pixel, estimated from the measured output voltage. The distribution reflects the photon flux effectively coupled to each pixel under controlled illumination conditions. g) Region‐dependent fluorescence signal patterns recorded across the 13‐shank geometry. Signals are grouped according to anatomically defined cortical regions (S1, PtA, and V2), demonstrating distinct spatial fluorescence dynamics across the probe.

To validate the in vivo performance of our neural probe, we conducted electrophysiological recordings and optical recordings across multiple cortical regions, including the somatosensory cortex (S1), parietal association cortex (PtA), and visual cortex (V2) in vivo (Figure 7a). For the electrophysiological recordings, we acquired both LFPs and APs, enabling characterization of distinct brain states. First, in both S1 and V2, we observed clear changes in brain oscillations across different isoflurane concentrations (Figure 7b,c), consistent with previous reports [64]. In particular, under deep anesthesia, LFP signals showed a reduction in the alpha‐band (8‐12 Hz) peak and an increase in theta (4‐8 Hz) in both S1 and V2 (Figure 7c). Second, to confirm physiological relevance of detected neural signals (LFPs and APs), we compared neural activity under dark conditions and during visual stimulation in the visual cortex (Figure 7d,e). Visual stimulation elicited sensory‐evoked responses, characterized by increased low‐frequency (< 10 Hz) LFP power and higher firing rates (Figure 7e), as described previously [65, 66]. Together, these experiments confirm the robust performance of our in vivo electrophysiological recording across brain regions and experimental conditions.

To further assess the optical detection capability of the probe in vivo, we performed optical recordings under the same experimental conditions. Controlled illumination patterns were applied to emulate calcium‐dependent fluorescence dynamics with precisely defined intensity levels. A baseline intensity of approximately 1.8 nWmm^−2^ and a maximum of 2.6 nWmm^−2^ were used, covering the typical fluorescence intensity range reported for GCaMP indicators (Figure 7f). [15] This range falls within the linear operating regime of the device (Section 2.6), ensuring that the recorded optical responses reflect intrinsic detector characteristics without saturation or nonlinear distortion. As shown in Figure 7g, distinct optical activity patterns were reliably recorded across three cortical regions (S1, PtA, and V2), demonstrating region‐dependent optical signal detection in vivo. The measured ΔF/F_0_ signals exhibited spatial variation consistent with light propagation and attenuation in brain tissue, confirming effective photon detection and photo‐pixel transduction under physiological conditions. The demonstrated detection sensitivity and linearity within the physiological fluorescence intensity range support the technical feasibility of future GCaMP‐based calcium imaging applications.

These results demonstrate that the proposed neural probe supports reliable multimodal data acquisition, enabling stable in vivo electrical and optical recordings across multiple cortical areas.

### 2.9 Mechanical Properties of Neural Probe

We developed a CMOS neural probe with 13 shanks, substantially exceeding conventional 1–4 shank designs and enabling broader spatial coverage and higher‐resolution, multi‐site recording [20, 32]. High shank counts demand uniform mechanical performance to avoid breakage, misalignment, and tissue overload during insertion. Variance of stiffness across shanks, especially at the edge of the CMOS chip, can induce non‐uniform deformation and trajectory deviation. Since the precise positioning of each shank within the targeted brain region is essential for acquiring reliable signals, the mechanical stability of our neural probe must be ensured. Excessive insertion force increases the risk of probe damage and amplifies local tissue stress. Therefore, each shank must balance sufficient stiffness for penetration with mechanical compliance to minimize invasiveness. In particular, adequate stiffness is required to traverse the dura mater, the dense, elastic meningeal layer covering the brain, and to reach deep targets without bending or mechanical deformation of the shank. Achieving optimal insertion forces is critical to limit tissue injury, attenuate immune responses, and preserve signal quality for accurate neural recording.

**FIGURE 8:**
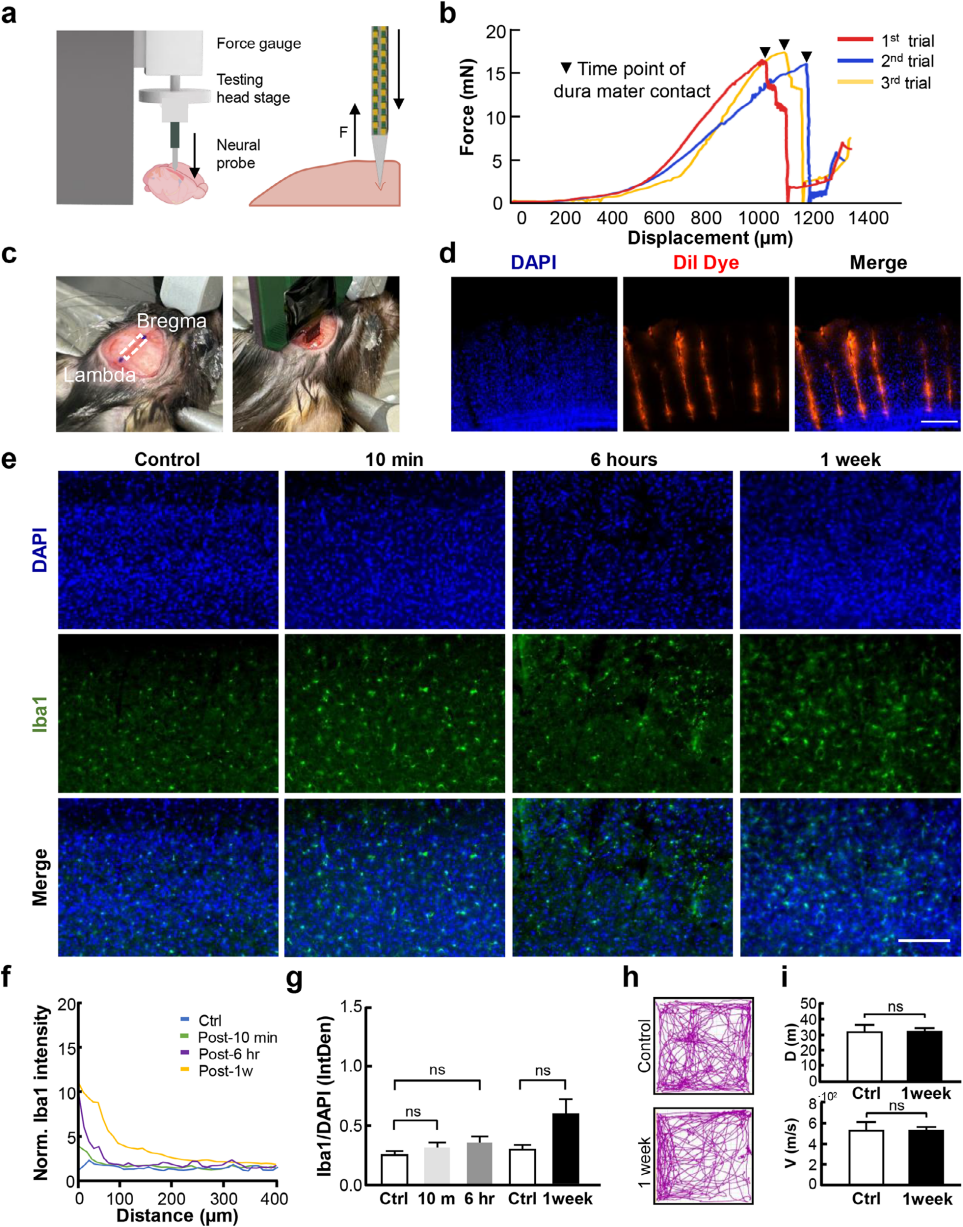
Tissue penetration force, neuroinflammatory responses, and biological safety evaluation of neural probe implantation. a) After the removal of the skull from the mouse brain, the neural probe was inserted with force gauge. b) The average insertion force by an individual shank was 17.1 mN. c) To ascertain the extent of neuroinflammation, a surgical procedure was performed on the mouse to insert the probe into the brain. The neural probe was slowly inserted in the sagittal direction targeting somatosensory and visual cortex (AP –0.4 mm, ML 1.5 mm, DV –1.0 mm). d) Trajectory of the neural probe was visualized by coating the probe shank with DiL (scale bar 275 µm). e) Immunohistochemical (IHC) analysis was performed to assess inflammatory responses at short and long‐term time points (scale bar 150 µm). Microglial activation was evaluated using Iba1 as an inflammation marker. f, g) Quantification of inflammatory responses following probe implantation. f) Normalized Iba1 fluorescence intensity was measured from the probe–brain interface to 400 µm, where values converged with control levels. Early time points showed a slight increase in peri‐probe intensity compared to control, while 1‐week post‐insertion exhibited a modest but spatially confined elevation that followed a similar distance‐dependent decline toward baseline. g) Iba1 intensity normalized to DAPI showed no statistically significant differences in either short or long‐term groups. Statistical significance was determined using one‐way ANOVA by Dunnett's post hoc test and unpaired two‐tailed t‐test (N = 2, tissue = 9–13, ns for non‐significance, p > 0.05). h) Open field test was conducted to evaluate motor deficits after 1‐week probe implantation. i) Total traveled distance (D) and mean locomotor speed (V) were analyzed and compared between the implanted (N = 1, 4 sessions) and control mice (N = 2, 4 sessions each). No significant differences were observed in either parameter, indicating that probe implantation for 1 week did not induce detectable motor deficits or impair spontaneous locomotor activity. Statistical significance was determined using an unpaired two‐tailed t‐test (ns for non‐significance, p > 0.05).

We performed a mechanical insertion force test using Mark‐10 force Gauge (Figure 8a, Figure S13) after removing the skull of the mouse brain and measured the average force required for each shank to penetrate the dura mater (Figure 8a). Due to its dense and elastic nature, the dura mater presents substantial mechanical resistance, and adequate stiffness is required to overcome this barrier without causing structural failure. The peak insertion force was measured in triplicate (n = 3 technical trials) for each condition and the average force was 0.222 ± 0.003 N, corresponding to an average per‐shank load of 17.1 mN assuming simultaneous engagement (Figure 8b). This load exceeded the shank's critical buckling requirement and was sufficient for dura mater penetration without observable mechanical failure, indicating that the CMOS chip supports safe, accurate targeting with minimal force and without buckling [67, 68]. These results highlight sufficient stiffness, compliance, and mechanical stability of the 13‐shank structural design that is necessary for high‐density implantation while preserving tissue compatibility during the insertion.

### 2.10 Short‐Term and Long‐Term Tissue Response Assessment

To evaluate the progression of tissue reactivity following short and long‐term probe implantation, the probe was inserted into the somatosensory and visual cortex – the same regions utilized for functional in vivo recordings (Figure 8c,d). Microglial activation was assessed by immunohistochemistry (IHC) using ionized calcium‐binding adapter molecule 1 (Iba1) at three time points: 10 min and 6 h (short‐term) and 1 week (long‐term) post‐insertion, with non‐inserted tissue as control (Figure 8e) [69, 70, 71, 72]. Normalized Iba1 fluorescence intensity was quantified as a function of distance from the insertion site (Figure 8f). At all‐time points, Iba1 expression was elevated near the insertion site and declined with increasing distance, consistent with a spatially confined foreign body response [71, 73]. The Iba1/DAPI intensity revealed no statistically significant differences between the short‐term groups (10 min and 6 h) and control (p = 0.5518, p = 0.1121, respectively), nor between the 1‐week group and control (p = 0.0663; unpaired two‐tailed t‐test) (Figure 8g). These results indicate that probe implantation did not elicit a significant inflammatory response relative to non‐inserted tissue across the examined time points.

### 2.11 Behavioral Assessment and Functional Integrity

Following probe insertion and head‐mounted maintenance for 1‐week, behavioral evaluation was conducted in parallel with the histological analysis to assess potential functional abnormalities. An open field test was performed to quantify spontaneous locomotor activity (Figures 8h, i). Total traveled distance and mean locomotor speed were measured and compared between the implanted (N = 1, 4 sessions) and control mice (N = 2, 4 sessions each). The results showed no significant differences in either metric, indicating preserved motor performance after implantation (p = 0.9817 for distance, p = 0.9783 for mean speed; unpaired two‐tailed t‐test).

## 3 Conclusion

This study introduces a chiplet‐based fabrication methodology that unifies the complementary strengths of research‐fab customization and commercial CMOS integration for neural probe development. By addressing the fundamental challenge of post‐CMOS processing on millimeter‐scale MPW chiplets through edge‐beading suppression and metal‐reinforced etch‐stop structures, our approach enables application‐specific multimodal neural interfaces that combine research‐fab design flexibility with commercial‐grade signal processing capabilities. The concept of edge‐beading suppression method using 3D printed frame was introduced to mitigate photoresist accumulation at the chiplet boundary, which is primarily governed by the photoresist flow dynamics, surface tension, and meniscus redistribution. EBSF modifies the photoresist flow boundary condition during the spin coating process; this concept is fundamentally geometry‐driven and therefore can be adopted with different resist chemistries and target photoresist thicknesses. In contrast, a multilayer metal etch‐stopping strategy is inherently material‐ and process‐dependent, as its effectiveness is governed by etch selectivity, self‐passivation behavior, over etching tolerance, and plasma chemistry. Even for the same nominal metal (e.g., Al‐based stacks), variations in alloy composition, barrier layers, and plasma conditions can alter stop‐layer robustness, requiring process‐specific validation. While our fluorine‐based etching provides sufficient selectivity for reliable Al‐based etch stopping, transferring this approach to alternative metal systems (e.g., different Al alloys, Cu‐based stacks, or modified barrier/capping layers) or different passivation schemes necessitates re‐optimization of both metal thickness and plasma chemistry.

The resulting 13‐shank probe, integrating 416 electrodes and 832 photodiodes with complete on‐chip signal processing, represents three notable firsts: (1) simultaneous high‐density electrophysiology and GECI‐based calcium imaging with active circuits, (2) more than eight independent shanks in an active CMOS probe, and (3) complete on‐chip analog‐to‐digital conversion for both electrical and optical modalities.

In vitro validation confirmed the stable and reliable operation of the proposed platform. More importantly, extensive in vivo experiments demonstrated robust and physiologically relevant multimodal recordings under realistic biological conditions. The device consistently captured both local field potentials and action potentials across multiple cortical regions and experimental states, including varying anesthesia levels and sensory stimulation paradigms. Concurrent optical recordings further validated the capability to detect spatially and temporally resolved fluorescence signals in brain tissue. Mechanical testing confirms insertion forces (0.222 ± 0.003 N) sufficient for dura penetration without buckling, while short and long‐term biocompatibility studies show minimal glial activation (no significant difference vs. control, p > 0.05). Following 1‐week implantation, no statistically significant differences in locomotion or mean speed were observed between implanted and control groups (p > 0.05), indicating preserved motor function.

Beyond these specific achievements, the broader significance lies in establishing a generalizable platform that preserves both customization advantages of research fabrication and the integration capabilities of commercial CMOS. Researchers can now engineer application‐specific electrode materials for optimized electrochemical sensing, design custom photodiode architectures for wavelength‐specific quantum efficiency, integrate novel sensing modalities (temperature, pH, neurotransmitters), or implement specialized on‐chip signal processing algorithms – all while retaining the proven circuit design methodologies, multi‐layer interconnect capabilities, and thousand‐channel scalability of standard CMOS foundries. This convergence of custom functionality with commercial integration density addresses a fundamental limitation where existing neural interfaces must compromise either design flexibility or recording scale. This platform particularly enables investigation of the 5,300+ molecularly‐defined cell types recently identified in mammalian brains – correlating their calcium dynamics with network electrophysiology to reveal how genetically‐distinct populations coordinate computations across brain regions, respond selectively to neuromodulators, or exhibit differential vulnerability in neurological disorders.

## 4 Experimental Section

### 4.1 3D Printing Parameters for the Fabrication of Edge‐Beading Suppression Frame

For the fabrication of edge‐beading suppression frame, we used a fused‐filament 3D printer (M160, Moment) with polylactic acid filaments with a total material usage of 519 mm^3^ (0.65 g). Schematic design of the frame was performed with TinkerCAD; 500 µm of height with 600 µm margin between the chiplet and the frame. Slicing parameters were: layer height 0.10 mm; 12 top and 5 bottom solid layers; 2 outline perimeters; first layer height 300% (0.30 mm), first layer width 100%, and first layer speed 50%. Infill was set to 50% with 100% infill extrusion width, 10% outline overlap, a 3 mm minimum infill length, and a 25 mm^2^ solid‐layer infill threshold area; the top‐layer extrusion modifier was 100%. Start points were optimized for fastest printing and restricted to preferred regions, yielding a dimensionally stable frame with smooth surfaces suitable for spin‐coating experiments. Additional details regarding the fabrication of edge‐beading suppression frame, including inner frame dimensional discrepancies and edge beading profiles at different frame thickness are provided in Figures S3 and S4.

### 4.2 Experimental Procedures and Chemicals for the Microfabrication of the Neural Probe Shank

All fabrication processes were carried out in a class 100 (ISO 5) cleanroom environment. Prior to SiO_2_ and front‐side Si etching, both the carrier wafer and CMOS chip were cleaned sequentially with acetone, isopropyl alcohol (IPA), and deionized (DI) water, followed by nitrogen gas drying. We used 4‐inch p‐type (100) silicon carrier wafer with a thickness of 500 µm and Si wafer with 2 µm of SiO_2_ by plasma‐enhanced chemical vapor deposition (PECVD) as a carrier wafer for SiO_2_ and Si etching process, respectively. After cleaning, photoresist bonding between the chip and the carrier wafer was performed using AZ GXR‐601 photoresist (Merck KGaA, Darmstadt, Germany) applied by spin coating. After bonding the neural probe, hexamethyldisilazane (HMDS) was coated onto the chip to enhance the adhesion between the chip surface and the photoresist layer, followed by spin coating for shank patterning (AZ 10XT 520cP, Merck KGaA, Darmstadt, Germany) (Table S4). During spin coating, an edge‐beading suppression frame (EBSF) was attached to minimize edge‐bead formation. Ramped soft‐baking step was conducted to evaporate residual solvents, and the sample was left at room temperature for approximately 20 min before photolithographic exposure (Table S5). Photolithography was carried out in hard‐contact mode (Karl Suss MA6 mask aligner), and for SiO_2_ and front‐side Si photoresist mask, we used MASK 1, as mentioned in Figure S7. After exposure, the wafer was left at ambient conditions for over an hour, followed by development and hard baking.

The SiO_2_ layer was etched using ICP dielectric etcher with CHF_3_ and Ar gas, and at an etch rate of 53 Å/sec (Table S3). Front‐side Si etching was subsequently performed using a deep trench reactive‐ion etching (RIE) system under C_4_F_8_, SF_6_, O_2_ at an etch rate of 0.5 µm/cycle (Table S3). After completing front‐side etching, the remaining photoresist and oxide residues were removed through standard cleaning. The CMOS chip was then flipped for backside processing, bonded again to the carrier wafer without additional patterning, and etched under identical conditions to define the final shank structure. Comprehensive post‐CMOS fabrication details are mentioned in Table S2.

### 4.3 Ti/Pt Deposition Onto Al Electrodes

After releasing individual shanks, 0.6 µm of Si_3_N_4_ and 3.75 µm of SiO_2_ layers on the Al electrodes were removed with reactive ion etching (RIE). The dielectric layer above the electrodes was opened with a 15 µm diameter window, and 2.4 µm of the metal at the electrode sites was exposed (Table S6). Subsequently, we spin‐coated the chip with negative DNR‐L‐300 (DONGJIN, KOR) at 4000 rpm for 30 s and exposed at the power of 182 mJ/cm^2^ with MASK 2 to pattern the photoresist mask for Pt deposition (Figure S7, Table S7). After development with the solution comprised of 2:1 (v/v) ratio of MIF 300 and DI water, we deposited 15 nm of Ti for enhancing the adhesion with Al and 100 nm of Pt using electron‐beam (E‐beam) evaporation and lifted off the metal at undesired sites.

### 4.4 Color Filter Fabrication and Deposition Process

Before the deposition of color filter onto the neural probe, we mixed 40 mg of ABS 473 absorptive dye (maximum absorbance: 473 nm, Luxottica Exciton, USA) and 1 mL of SU‐8 2002 photoresist (MicroChem Corp., Massachusetts) [15, 51] to utilize powder form of absorbing dye as a color filter and stirred vigorously with vortex mixer, followed by 20 min of sonication. The dye concentration (4% (w/v)) and filter thickness (2.54 µm) were optimized to maximize the transmittance ratio of emission‐to‐excitation light, achieving strong rejection of 480 nm excitation while maintaining >30% transmission at 520 nm emission wavelengths [15]. After the preparation of color filter, we attached the chip onto the carrier wafer and deposited HMDS and color filter by spin coating process at a rate of 3000 rpm (Table S8). After soft baking, we applied UV exposure at a power level of 2640 mJ/cm^2^ to cure the color filter with color filter Mask 3 (Figure S7) and developed with SU‐8 developer for 1 min 30 s. To accommodate potential alignment errors during photolithography, the color filter mask was intentionally oversized to ensure complete photodiode coverage while avoiding electrode regions.

### 4.5 Gap Measurement Method and Photoresist Thickness Measurement

To optimize the edge beading suppression using a modified spin coating process and the edge‐beading suppression frame (EBSF), the gap between the chiplet and the frame was quantified by optical microscopy, and the photoresist thicknesses were measured with a profilometer (Alpha step IQ, KLA‐Tencor).

### 4.6 Focused Ion Beam and Scanning Electron Microscopy (SEM)

Focused ion beam (FIB) milling (Nova 600 NanoLab, FEI) was performed with accelerating voltages of 5–30 kV using Ga ions to analyze the cross‐section of recording channels and electrode‐photodiode array. Scanning electron microscopy (SEM) images were acquired using a field‐emission SEM (Inspect F, FEI) with a Schottky field emission source.

### 4.7 In Vitro Characterization Setup

For the optical property measurement, a photodetector (818‐SL/DB, Newport Inc., Irvine, CA, USA) was coupled to an optical power meter (1936‐R, Newport Inc., Irvine, CA, USA). The input light from a xenon lamp (ASB‐EX‐175, Spectral Products, Putnam, CT, USA) was wavelength‐selected through a monochromator (CM110, Spectral Products) and then coupled into an integrating sphere (819D‐IS‐5.3, Newport Inc., Irvine, CA, USA) equipped with a photodetector to provide isotropic illumination to the probe and simultaneously monitor the optical power as wavelength changed. Monochromatic light ranging from 400 to 700 nm was applied to the probe in 10 nm intervals to analyze the spectral responses of color filter (Figure 5a). In Figure 5b,c, excitation and emission wavelengths were specifically set to 480 nm and 520 nm, respectively, and the output intensity of the xenon lamp was adjusted to control the optical power incident on the probe. For the photo‐pixel characterization, the wavelength was fixed at 520 nm, and the output response was measured while varying the incident light intensity (Figure 6a). In the dark noise characterization process, the xenon lamp was turned off, and the photo‐pixel output was recorded under ambient conditions in which the power meter reading (520 nm setting) remained around a baseline offset of −1.04 pW (Figure 6b).

Electrical characterization of the recording circuit was performed using a function generator as the input source (33622A, Keysight Technologies, Santa Rosa, CA, USA). To evaluate the ADC bandwidth, a 10 mV_pp_ sinusoidal signal sweeping from 10 Hz to 9 kHz was applied, with ten frequency points per decade (Figure 6e). The noise spectral density was measured with the electrode input shorted to the reference electrode to obtain the intrinsic readout noise (Figure 6f). The chip was powered by a DC power supply (E36311A, Keysight Technologies, Santa Rosa, CA, USA) and a precision DC voltage source (DC205, Stanford Research Systems, Sunnyvale, CA, USA), providing 5 and 1.8 V supply rails and a 2.5 V bias for the photodiode, with all voltage sources sharing a common ground. For digital interfacing and data acquisition, the chip was mounted on a custom PCB and connected to an FPGA (Opal Kelly XEM7310‐A200). Digital control signals were applied based on a synchronized clock domain, and output codes were acquired within the same clock domain to ensure timing consistency during readout.

Device‐induced temperature rise was evaluated using a thermal imaging camera (FLIR E5 Pro, Teledyne FLIR, USA). Measurements were performed under two conditions: (i) power‐off baseline with no input signals and (ii) power‐on & recording state with active power supply and simultaneous readout from electrodes and photodiodes. All 13 shanks were monitored continuously for over 10 min to ensure that thermal equilibrium was reached prior to analysis. Temperature changes were quantified by comparing the steady‐state temperature in the recording condition to the baseline state. Measurements were conducted in air under ambient laboratory conditions.

### 4.8 In Vivo Electrical and Optical Recording

Stereotaxic implantation of our neural probe was performed at the somatosensory and visual cortex regions. A 10‐week‐old C57BL/6 wild‐type female mouse (N = 1; KIST, Republic of Korea) was anesthetized with isoflurane (3%–5% for induction, 1–2% for maintenance) and secured in a stereotaxic frame (Kopf Instruments, USA). All procedures were approved by the Korea Institute of Science and Technology (KIST, Seoul) Institutional Animal Care and Use Committee (IACUC) and were conducted in accordance with the Animal Care and Use Guidelines. Following scalp incision, a sagittal‐oriented craniotomy was performed by removing the skull overlying both the somatosensory and visual cortex (anteroposterior, AP: –0.4 mm; mediolateral, ML: 1.5 mm relative to bregma). Two stainless‐steel screw electrodes were implanted over the cerebellar skull (AP: –1.5 mm; ML: ±1.8 mm relative to lambda) for ground and reference. The dura mater was carefully removed to facilitate atraumatic probe insertion. To minimize electrical noise, the stereotaxic frame with the head‐fixed mouse was positioned inside a grounded metal chamber. The probe was slowly advanced to a depth of dorsoventral (DV) –1.1 mm and subsequently retracted by 0.1 mm while monitoring the insertion process under a surgical microscope. The exposed cortical surface was continuously kept moist with saline‐soaked Gelfoam.

Electrophysiological and optical recordings were performed sequentially. For light anesthesia recordings, the isoflurane concentration was reduced to 1.0% and recordings were initiated after a 30‐min stabilization period. Recordings were obtained from shanks 1 to 13 with each recording lasting at least 30 s. For deep anesthesia recordings, the isoflurane concentration increased to 1.5% followed by a 10‐min stabilization. Recordings were again obtained from shanks 1 to 13 for at least 30 s each. To assess visually evoked responses, flash light stimulation was delivered to the contralateral eye under deep anesthesia, and neural activity was recorded for at least 1 min. For optical recordings, an LED source was positioned at the cortical surface to deliver temporally emulated, fluorescence‐like optical signals. The incident illumination was calibrated to a baseline intensity of 3 nWmm^−2^ with a peak of 4.5 nWmm^−2^ at the light source. The effective light intensity estimated at the photo‐pixel ranged from approximately 1.8 to 2.6 nWmm^−2^, reflecting attenuation during photon propagation through brain tissue due to scattering and absorption. After completion of recordings, the probe was slowly retracted.

### 4.9 Mechanical Force Testing of Neural Probe

A mechanical insertion force test was performed with Mark‐10 Force Gauge (Figure 8a; Figure S13) after removing the skull of the mouse brain (Figure 8a). Wire‐bonded neural probe was securely attached with 3D‐printed holder on the testing head of the instrument (Figure S13). The average force for each shank to penetrate the dura mater was measured at a speed of 1.1 mm/min.

### 4.10 In Vivo Analysis of Inflammation Induced by Probe Insertion

Evaluation of tissue response under physiological conditions is essential for establishing probe biocompatibility and for verifying that the shank's mechanically optimized elasticity and stiffness minimize neuroinflammation. Stereotaxic implantation was performed (Kopf Instruments, USA) of our neural probe into the somatosensory and visual cortex region. C57BL/6 wild‐type male mice (N = 2, 10 weeks old; DBL, Republic of Korea) were anesthetized with isoflurane (3 –5% for induction, 1–2% for maintenance) and secured in a stereotaxic frame (Kopf Instruments, USA). All procedures were approved by the Korea Institute of Science and Technology (KIST, Seoul) Institutional Animal Care and Use Committee (IACUC) and conformed to the Animal Care and Use Guidelines. After scalp incision, a craniotomy was made along the sagittal direction by removing the skull overlying both the somatosensory and visual cortex (anteroposterior, AP: −0.4 mm; mediolateral, ML: 1.5 mm relative to bregma). The dura was carefully removed to facilitate low‐trauma insertion. The probe was advanced slowly to a depth of dorsoventral (DV) –1.0 mm. To verify the insertion trajectory, the probe shank was coated with DiL dye prior to implantation, and the fluorescent track was used to confirm the insertion region. Trajectory imaging was performed in a separate mouse from histological analysis to avoid interference between DiL labeling and staining.

After securing the head and identifying the target coordinates, the probe was slowly inserted into the brain (1.0 mm/min). After a 10‐min stabilization period, the chip was inserted according to the designated short‐term (10 min and 6 hr) and long‐term (1‐week) time points, and then slowly withdrawn at a rate of 1.0 mm/min to assess tissue impact.

Subsequently, immunohistochemistry (IHC) was performed to determine whether the Si shank induced inflammation during the penetration following established protocols [74]. Ionized calcium‐binding adapter molecule 1 (Iba1) was selected as a marker of microglia activation. Following probe insertion, mice were sacrificed and brains were collected. For fixation, brains were placed in 4% paraformaldehyde (PFA) at 4 °C overnight, followed by dehydration with 30% sucrose for 2 days. Brains were sectioned with cryostat CM1800 (LEICA biosystems, IL, U.S.A) at 40 µm and blocked in PBT (0.03% Tween‐20 in PBS) with 0.3% Triton X‐100 and 6% donkey serum for 1 h. Brain slices were incubated with goat anti‐Iba1 (1:200, Abcam, ab5076) primary antibodies. After washing, slices were treated with Alexa Fluor 488‐conjugated donkey anti‐goat (1:500, Sigma–Aldrich, A11055) for 1 h. Samples were mounted using fluorescence mounting medium and imaged with an EVOS M7000 microscope, and the fluorescence intensity was analyzed using ImageJ (National Institutes of Health, MD, USA).

### 4.11 Behavior Analysis

An open field test was performed in the behavioral testing room. The neural probe was mounted with dental cement for 1‐week on a C57BL/6 wild‐type male mouse (10 weeks old), with two age‐matched littermates serving as controls (Figure S14). Locomotion and mean speed were analyzed using the Anymaze video tracking system (Stoelting, USA) and a digital camera. The open field test was performed according to the previous studies [75]. Each mouse was placed in the center of a white chamber (40 cm × 40 cm × 50 cm) with 20 min acclimatization. After 1 day of acclimatization, each mouse was recorded for 10 min per session (4 sessions per mouse) to evaluate total traveled distance and mean speed.

### 4.12 Statistical Analysis

Statistical analysis was performed with one‐way ANOVA with Dunnett's post hoc test for multi‐group comparison, paired t‐tests for within‐subject comparison, and unpaired two‐tailed t‐test for between‐group comparison, using GraphPad Prism software (GraphPad Prism Software Inc., version 10). Data are presented as mean ± standard error of the mean (S.E.M.). Data preprocessing, quantitative analysis, and figure generation were performed using custom MATLAB scripts (MathWorks, R2023b).

## Author Contributions

J.H.M., M.K., and C.L. conceived and designed the research. C.L. and M.K. designed the integrated circuits. J.H.M. performed post‐CMOS fabrication and optical packaging. K.K. assisted with initial back‐end process development. M.K. designed the electronic control system and interface, and performed system‐level characterization and signal processing analysis. J.H.M. performed surgical procedures and immunohistochemical analysis. W.S. and Y.P. contributed to the design and execution of in‐vivo validation experiments and assisted with data visualization and manuscript preparation. S.L. advised on fabrication process development and contributed to figure design and manuscript writing. J.K. co‐designed and supervised the in‐vivo experiments. J.H.M., M.K., S.L., and C.L. wrote the manuscript. All authors discussed the results and commented on the manuscript. C.L. conceived the project and provided overall supervision. I.‐J.C., M.S.K., J.W.S., and J.K. provided supervision.

## Conflicts of Interest

The authors declare no conflicts of interest.

## Supporting information




**Supporting File**: advs74850‐sup‐0001‐SuppMat.docx.

## Data Availability

The data that support the findings of this study are available from the corresponding author upon reasonable request.
