# Supplementary material for: Fabrication of High‐Density Multimodal Neural Probes Based on Heterogeneously Integrated CMOS
Source: Adv Sci (Weinh). 2026 Mar 24;13(34):e24260. doi: 10.1002/advs.202524260 (PMC13285171; doi:10.1002/advs.202524260)
Supplement: Supplementary file 1 — Supporting File: advs74850‐sup‐0001‐SuppMat.docx. [file ADVS-13-e24260-s001.docx]

**Supporting Information:**

**Fabrication of High-Density Multimodal Neural Probes Based on Heterogeneously Integrated CMOS**

Ju Hee Mun^1,2†^, Miji Kim^1,3†^, Wooyeon Shin^1^, Yongjun Park^1,4^, Kanghwan Kim^1^, Il-Joo Cho^5^, Jae Won Shim^2^, Min Soo Kim^1,4,6^, Sunwoo Lee^7^, Jeongjin Kim^1,4^, Changhyuk Lee^1,8*^

*^1^Brain Science Institute, Korea Institute of Science and Technology (KIST), Seoul 02792, Republic of Korea*

*^2^School of Electrical Engineering, Korea University, Seoul 02841, Republic of Korea*

*^3^Department of Electronic Engineering, Hanyang University, Seoul 04763, Republic of Korea*

*^4^Division of Bio-Medical Science & Technology, KIST School, University of Science and Technology, Seoul 02792, Republic of Korea*

*^5^Department of Biomedical Sciences College of Medicine, Korea University, Seoul 02841, Republic of Korea*

*^6^KHU-KIST Department of Converging Science and Technology, Kyung Hee University, Seoul 02447, Republic of Korea*

*^7^School of Electrical & Electronic Engineering, Nanyang Technological University, Singapore 639798, Singapore*

*^8^KIST-SKKU Brain Research Center, SKKU Institute for Convergence, Sungkyunkwan University, Suwon 16419, Republic of Korea*

*Corresponding author: changhyuk@kist.re.kr

^†^These authors contributed equally to this work

**Supplementary Note S1: Optimization of Three Sequential Etching Process**

The 14 μm photoresist thickness requirement derives from three sequential etching operations, each performed with fresh photoresist coating: (1) Front-side BEOL dielectric removal, (2) Front-side silicon trenching, and (3) Back-side silicon thinning. Reactive ion etching (RIE) removes 6-10 μm of CMOS back-end-of-line (BEOL) passivation, which consists of SiO2 and Si3N4 layers, using CHF3/Ar chemistry (**Table S3**). The achievable etch selectivity of SiO2 to photoresist in fluorocarbon RIE typically ranges from 0.4 to 8, depending on process conditions.[1,2] In our silicon oxide etching process, the measured etch selectivity was approximately 1.4. Scaling the back-end-of-line (BEOL) to support a higher number of channels with a multi-layer metal stack (M1-M6) resulted in an increased oxide dielectric thickness, with focused ion beam (FIB) cross-sectional measurements showing a thickness of ~11.12 ± 0.05 μm; accordingly, a target etch depth of 12 µm was specified to accommodate local variations and a 10 % over-etch, ensuring complete exposure of silicon layer. A 14-µm-thick photoresist layer was applied to ensure a ≥ 2-3 µm residual margin at the target depth, yielding a nominal front-side dielectric etch depth of 13.2 µm measured from the dielectric surface to the exposed silicon interface (**Figure S2**).

After removing SiO2 layer between shanks (**Figure 3a, d**), front-side deep reactive ion etching (DRIE) using the Bosch process removes silicon to define individual probe shanks and the back-side DRIE removes bulk silicon to achieve final shank thickness and the complete shank release (**Figure 3b, d**). The target values were determined by calibrating the actual etch depth relative to the nominal value under the standard Bosch process (**Table S3**). In the calibration run, the front-side etching removed about 73.5 μm of Si (~74 % compared to 100 μm target depth), and the remaining Si substrate was 230 μm. Considering that our DRIE process consistently removes about 74 % of the programmed depth, the target etch depths were adjusted to ensure complete shank release while preserving sufficient base thickness for mechanical robustness. Accordingly, the front- and back-side Si etching depths were set to 180 μm and 280 μm, respectively, which provided complete shank release from the bulk substrate. Under these conditions, the resulting shank thickness was approximately 90 μm, remaining above the mechanical fragility threshold.

**Supplementary Note S2: Edge-Beading Index (EBI) Quantification**

We quantified edge-beading severity using the edge-beading index (EBI), defined as:

$$\boldsymbol{EBI (\%) = [(}\mathbf{h}_{\boldsymbol{edge}}\mathbf{-}\mathbf{h}_{\boldsymbol{center}}\mathbf{) /}\mathbf{h}_{\boldsymbol{center}}\boldsymbol{] \times100 (Equation S1)}$$

where hedge and hcenter are the photoresist thickness at chiplet edge and center, respectively (**Figure 2a**). For high-fidelity photolithography of 1-2 μm features, EBI should be maintained below ~50 % to ensure adequate depth of focus and pattern transfer quality. Initial spin coating with process optimization yielded EBI = 162 % (19.4 μm edge / 12 μm center; **Figure S3**), which was still insufficient for our thick photoresist strategy. The EBSF approach achieved EBI = 50 % (6.0 μm edge / 12 μm center; **Figure 3d-f**), meeting the threshold requirement.

**Supplementary Note S3: Spin Coating Parameter Optimization**

Several spin coating parameters (e.g., spin speed, acceleration profile) and types of photoresist control the photoresist thickness and uniformity.[3] As a first trial, we introduced a low-viscosity photoresist (46 cP) using manufacturer-recommended protocols; however, the initial coating exhibited severe edge beading (>28 µm at the die perimeter; **Figure S5a**). We then modified the spin parameters (spin speed/acceleration) to suppress the edge beading. Although this mitigated bead formation, it also reduced the **mean photoresist thickness to ~6 µm**, below the target thickness. To achieve the desired thickness, we switched to a **higher-viscosity photoresist (520 cP)** with the supplier protocol; this increased the nominal film thickness, but **exacerbated edge beading** flowed inward toward the chip center, forming an excessively thick photoresist coating overall (~88.9 µm; **Figure S5**). Notably, an excessively thick photoresist layer is not intrinsically advantageous for fine photolithography on CMOS dies. The longer optical path during UV exposure increases refraction and bulk scattering, leading to lateral light diffusion and reduced aerial-image contrast, effects that become particularly detrimental in high-resolution or densely patterned regions. To mitigate these effects, the first and second spin speeds as well as the acceleration profile were optimized (**Table S4**). The optimized conditions yielded a photoresist thickness of approximately 12 µm at the center, closely matching the target value, and the calculated maximum EBI (%) was 162 % (hedge – hcenter = 19.4 μm / 12 μm center thickness; **Figure S5b**). This parameter-based optimization alone was insufficient to fully address the issue; EBI of 162 % exceed depth-of-focus tolerance for contact lithography of 1-2 μm features.[4] This demonstrates that conventional parameter optimization alone is insufficient for chiplet-scale coating uniformity, necessitating the EBSF approach described in the main text.

**Supplementary Note S4: EBSF Design and Placement Optimization**

To address the geometric limitations, we developed edge-beading suppression frame (EBSF) with elevated frame boundary reduces surface-tension-driven meniscus formation at chiplet edges by providing a nearby vertical surface that preferentially wets the photoresist.[5,6] To determine the optimal EBSF height, we fabricated frames with target heights of 300, 400, 500, and 600 µm using a fused-filament 3D printer (M160, Moment) with 100 µm layer height resolution. The measured heights showed varying discrepancies from target values: 20.7 µm for 300 µm target, and 47.5 µm for 600 µm target, while the 400 µm and 500 µm designs were fabricated most closely to their intended dimensions (**Figure S3a**). Photoresist edge-beading profiles were characterized for each EBSF height condition (**Figure S3b**). The 500 µm EBSF (measured height: 508.8 ± 3.3 µm) provided optimal suppression, achieving EBI = 50 % compared to 162 % without EBSF. For the 330 µm thick CMOS chip, this corresponds to a height difference of Δh = 178.8 ± 3.3 µm, which positions the frame surface above the chip edge and redirects photoresist flow away from the chip boundary during spin coating. Ideally, the printed frame opening would match the chip dimensions exactly, resulting in zero gap. However, this is not mechanically feasible. While the CMOS chip dimensions showed negligible variation (<±10 µm), the 3D-printed frames exhibited substantial inward deviations: 165–240 µm along the width, 145–225 µm along the length (**Figure S4a**) and 205–245 µm corner rounding offset (**Figure S4b**). The corner rounding represents the dominant constraint on mechanical clearance. Given this practical resolution, a margin of 300 µm per side was selected to satisfy minimum required clearance. For center-aligned placement, this provides 600 µm total clearance per axis, exceeding the worst-case corner rounding offset. The gap was measured as the perpendicular distance from the chip edge midpoint to the corresponding frame inner edge using optical microscopy. For our probe design where functional structures occupy only the left half of the chip, corner-contact alignment was employed to minimize the gap at the probe region. The measured gaps under this configuration were–Lower-left region: 96 ± 3 µm (left edge, n = 5) and 57 ± 6 µm (bottom edge, n = 5), Upper-right region: 260.25 ± 5.65 µm (right edge, n = 5) and 378 ± 8.03 µm (top edge, n = 5). Prior studies indicate that photoresist viscous flow promotes bridging across gaps smaller than ~100 µm during spin coating.[7,8] The measured gaps at the lower-left corner (57–96 µm) fall within this regime, explaining the effective edge-bead suppression observed (**Figure S4c**). For applications where functional structures span the entire chip area, center alignment with the 300 µm nominal margin on all sides is recommended.

An oversized frame geometry creating controlled gaps was then evaluated in two configurations: (1) chiplet positioned in contact with frame lower-left corner, and (2) chiplet centered within frame, creating four symmetric gaps (**Figure 2c, S6**). Edge-beading thickness at left side, cross section, and under side was substantially lower with corner-contact configuration. The frame was aligned at the lower-left corner with average margin of 0.096 ± 0.003 mm (left) and 0.057 ± 0.006 mm (under side) to accommodate placement tolerance (**Figure S6**). Corner-contact alignment reduced residual gap area by 66 % versus center alignment, contributing to the 3.2× EBI improvement demonstrated in the main text.

**Supplementary Note S5: Post-Coating Baking and Development Protocols**

To accommodate thick photoresist and suppress voids from rapid solvent outgassing, ramped soft baking was performed (detailed conditions in Table S5). Rehydration delays of ~20 minutes were implemented both between soft baking and photolithography, and between photolithography and development. For positive diazonaphthoquinone (DNQ)/novolac photoresist, photo-generated acidic species continue reacting transiently after exposure; permitting this interval sharpens dissolution contrast.[9] Concurrently, delays promote uniform rehydration and relax residual stress, yielding improved developer penetration and selectivity.[10,11] Hard baking increased adhesion for subsequent etching while minimizing thermal distortion.


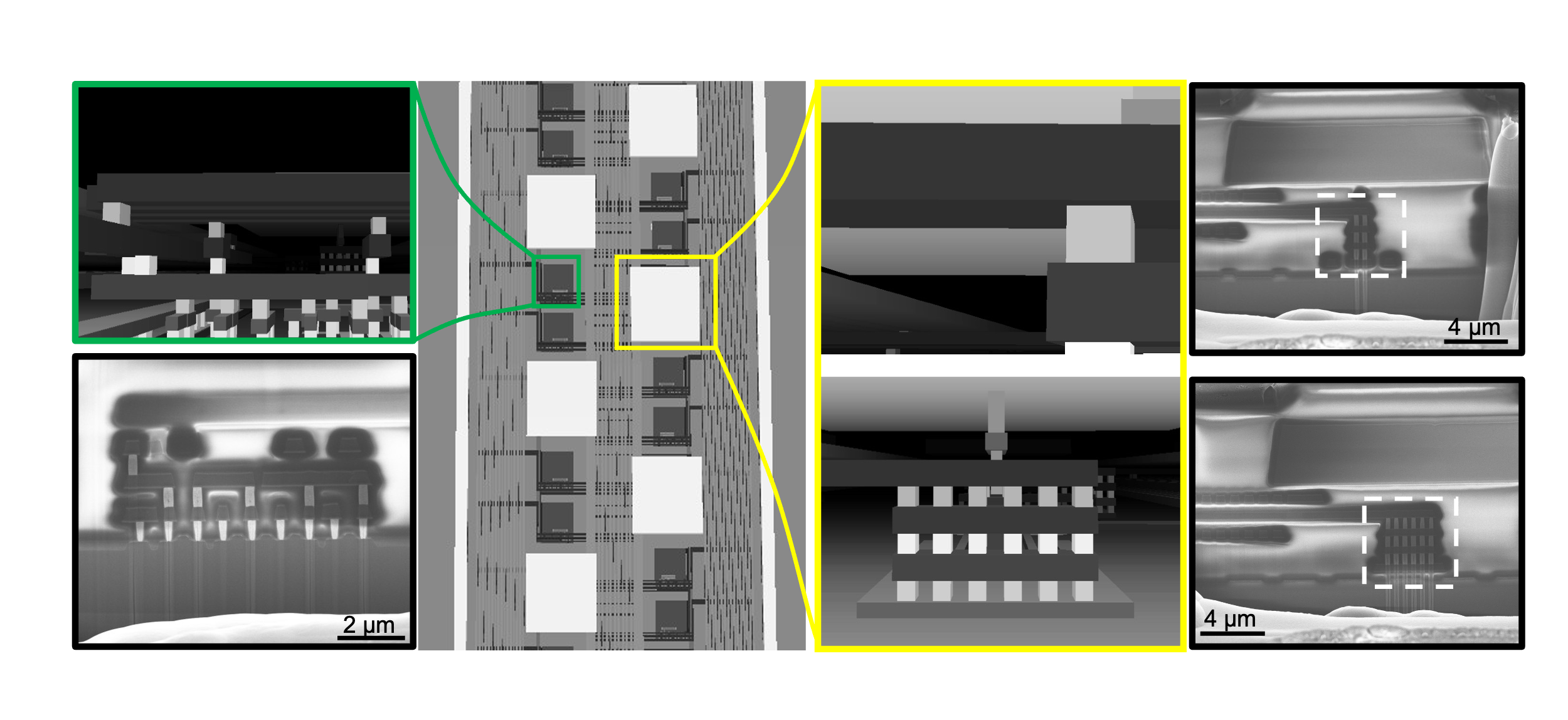
**Figure S1 |** 3D schematic layout and FIB cross-section validating multi-layer metal routing. The photodiodes (green box) and electrodes (yellow box) are realized using a continuous M1–M6 stack specified in the GDS 3D circuit layout; focused ion beam (FIB) cross-sections (black box) directly confirm this implementation. This physical validation shows that our metal-reinforced perimeter-wall strategy provides a robust, manufacturable pathway for scaling beyond the wiring constraints of passive multi-shank probes.


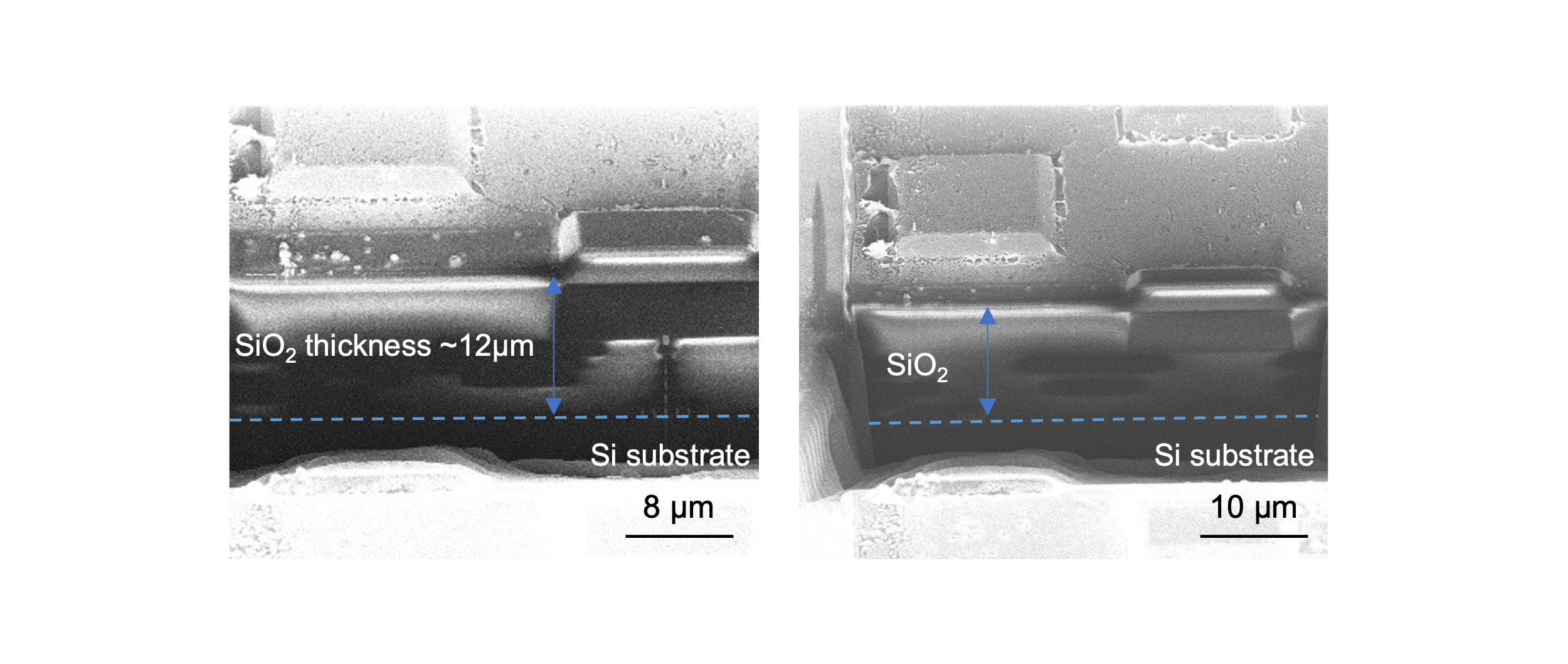
**Figure S2 |** FIB cross-sectional analysis of BEOL dielectric and Si substrate for oxide etching optimization. Focused ion beam (FIB) cross-sectional imaging was employed to precisely characterize the back-end-of-line (BEOL) dielectric layer thickness and identify the interface with the underlying Si substrate. The FIB images revealed a total BEOL dielectric thickness of approximately 11.2 µm, comprising intermetal dielectric (IMD) layers and passivation films deposited during standard CMOS processing. To ensure complete removal of the dielectric layer and reliable exposure of the Si substrate for subsequent deep reactive ion etching (DRIE) of the neural probe shanks, the oxide etching process was designed with a target depth of 12 µm with 10 % over-etching to compensate for process non-uniformities and full dielectric clearance without any residual oxide interferences.


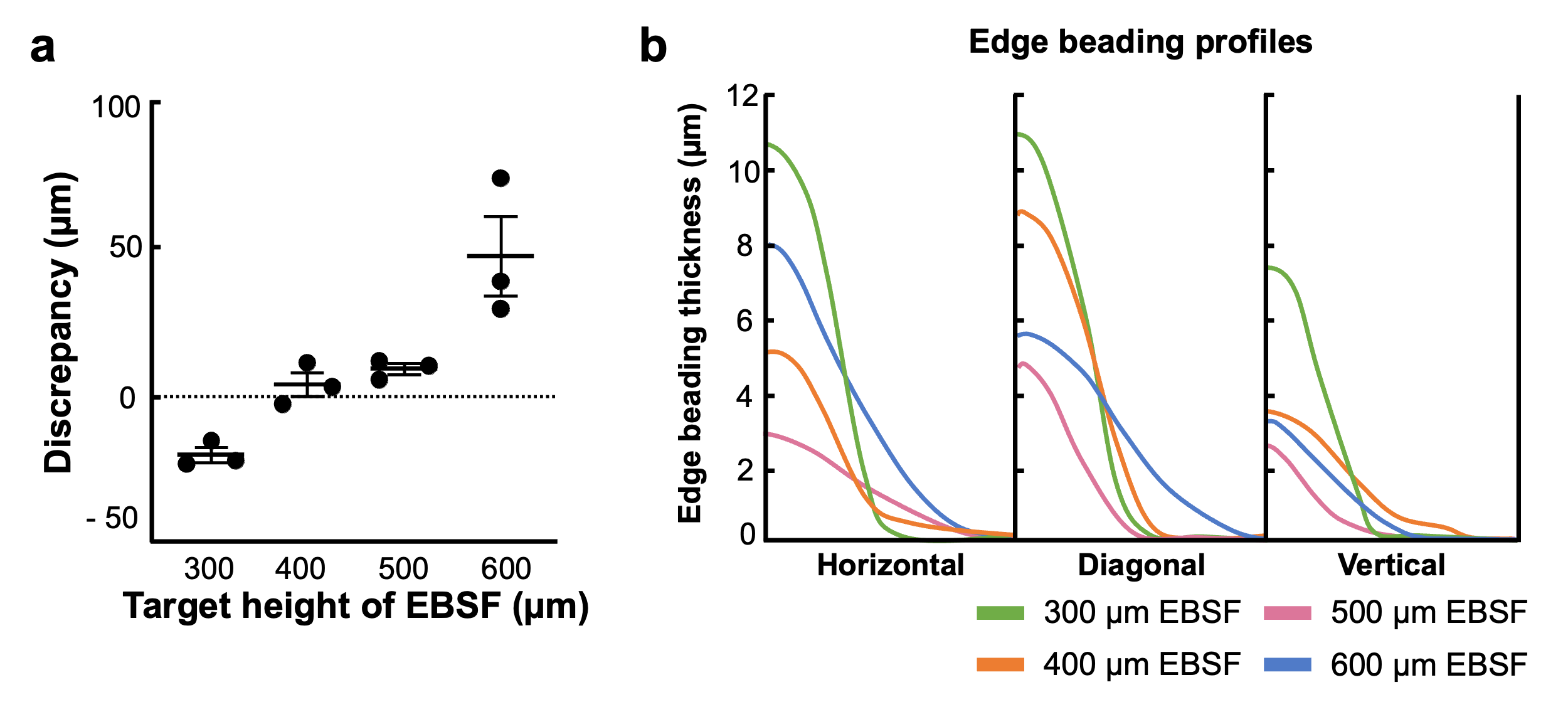


**Figure S3 |** Thickness-dependent dimensional accuracy and edge-beading suppression performance of 3D-printed EBSF with thickness variation. To characterize fabrication variability and process performance, edge-beading suppression frames (EBSFs) with different nominal thicknesses were fabricated using the same 3D printer and settings. a) We designed EBSFs with nominal heights ranging from 300 µm to 600 µm in 100 µm increments and compared the target heights with the actual fabricated heights. For the 300 µm and 600 µm designs, the discrepancies from the target height were relatively larger (20.7 µm and 47.5 µm, respectively), whereas the 400 µm and 500 µm EBSFs were fabricated most closely to their intended dimensions. b) Edge-beading profiles for all four EBSFs heights were analyzed from planarized center to edge-beading peak. 500 µm height of EBSF showed the maximized edge-beading suppression effect. Given the chip thickness of 330 µm, this corresponds to a nominal height difference of Δh_nom_ = 170 µm, and an experimentally measured height difference of Δh_meas_ = 178.8 ± 3.3µm.


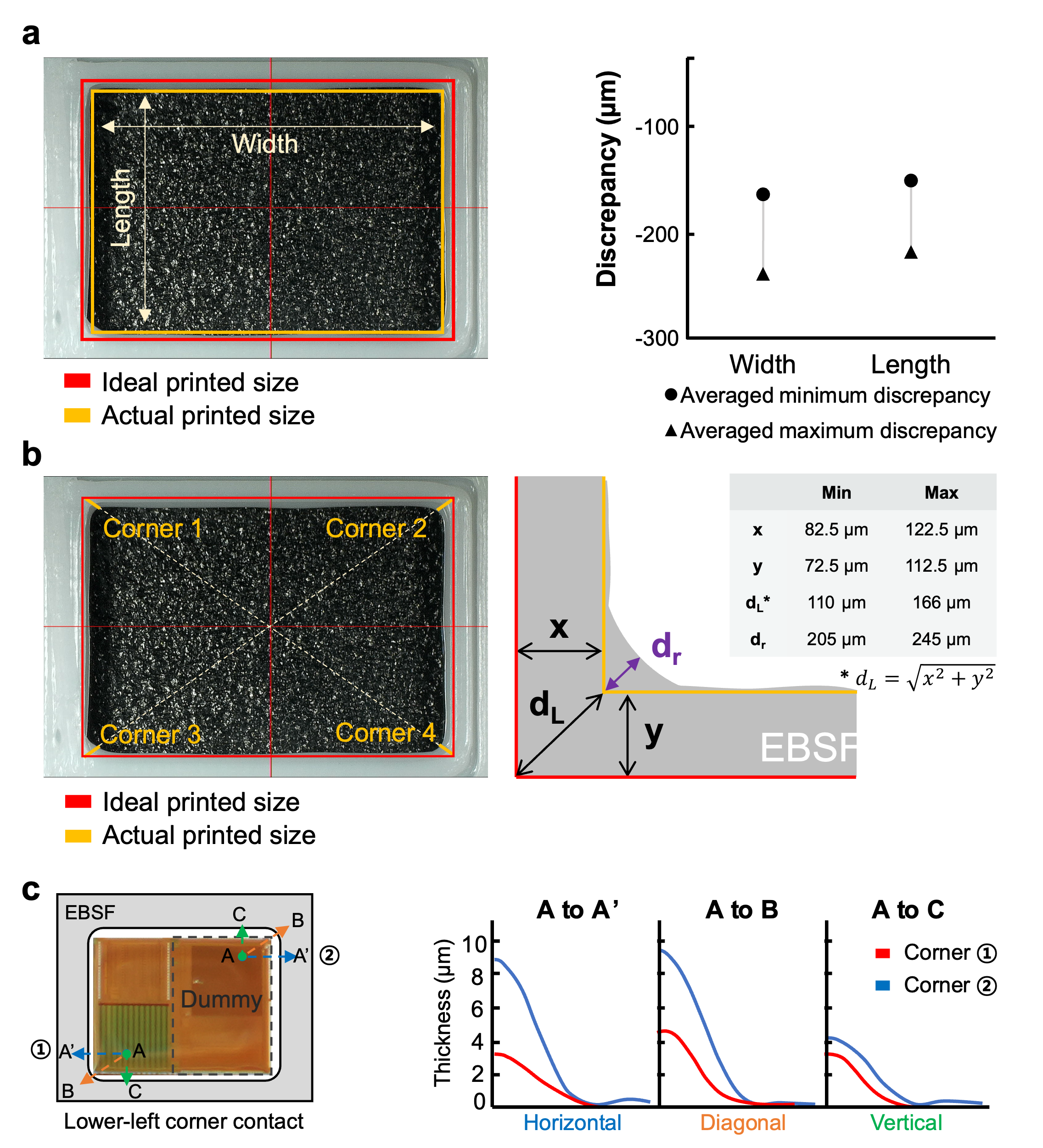


**Figure S4 |** Dimensional characterization of the inner geometry of the EBSF. a) The inner dimensions of the fabricated edge-beading suppression frame (EBSF) were evaluated to assess printing accuracy. The printed frames showed large deviations from the target dimensions, with inward discrepancies of 165–240 µm in width and 145–225 µm in length (batch = 2). b) The printed frame corners also exhibit finite rounded fillets rather than sharp right angles, resulting in a rounding-induced offset of maximum 411 µm into the chip corner regions (batch = 2). c) Edge-beading profiles at the lower-left and upper-right chip corners when the EBSF is aligned by lower-left corner contact. The lower-left corner (reference contact point) shows minimal edge-beading: 96 ± 3 µm (left edge, n = 5) and 57 ± 6 µm (bottom edge, n = 5), whereas the upper-right corner exhibits amplified edge-beading variation: 260.25 ± 5.65 µm (right edge, n = 5) and 378 ± 8.03 µm (top edge, n = 5), due to the increased local gap between the EBSF and the chip.


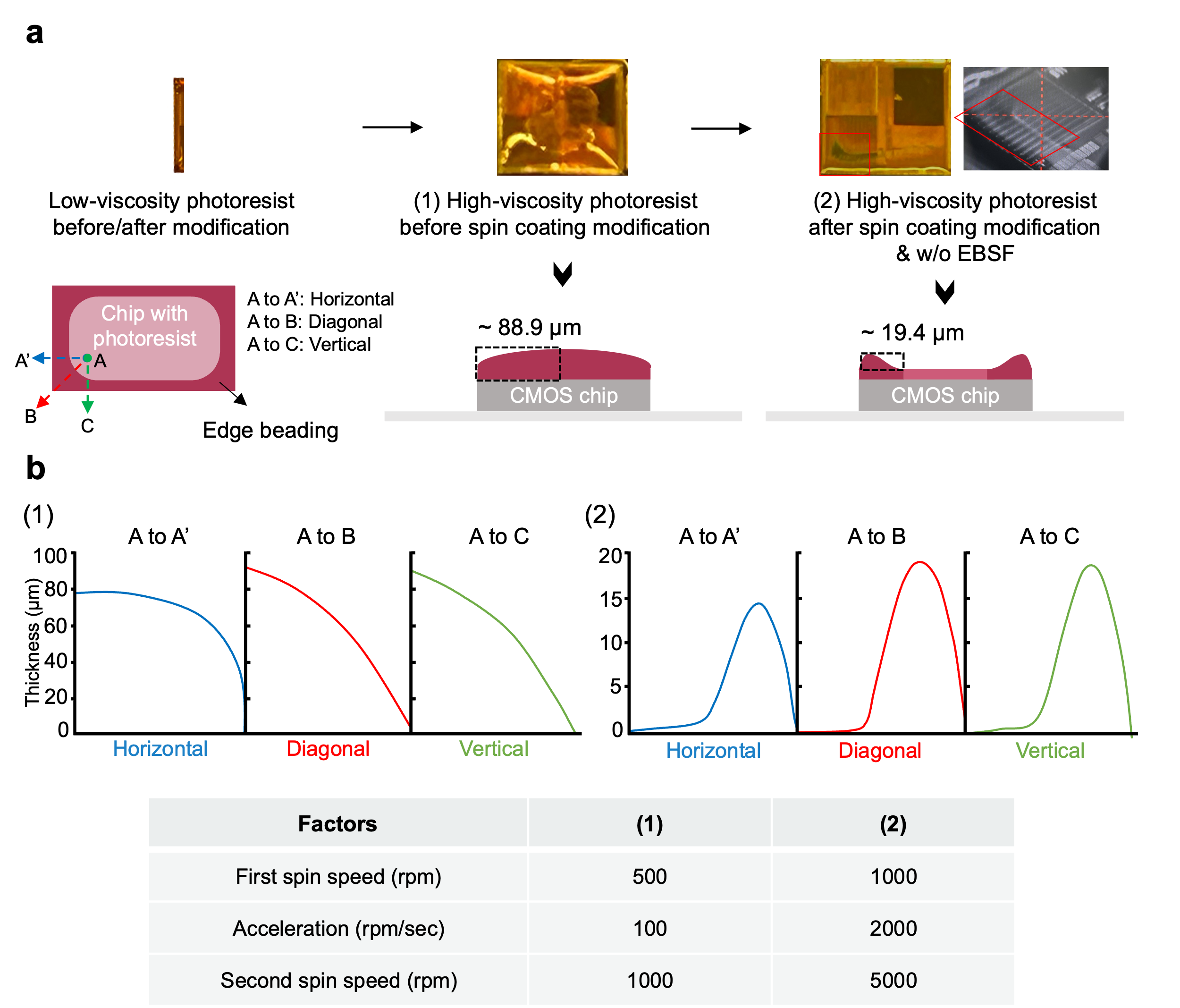


| **Factors** | **(1)** | **(2)** |
| --- | --- | --- |
| First spin speed (rpm) | 500 | 1000 |
| Acceleration (rpm/sec) | 100 | 2000 |
| Second spin speed (rpm) | 1000 | 5000 |

**Figure S5 |** Spin coating optimization to suppress edge-beading in chiplet-scale photoresist patterning. a) Low-viscosity photoresist following standard protocols produced severe edge-beading (left), while modified parameters reduced edge-beading but yielded insufficient thickness for deep etching (right). b) High-viscosity photoresist optimization. (1) Manufacturer's standard recipe achieved uniform coverage but induced excessive edge-to-center capillary flow due to small die dimensions (6.5 × 5.0 mm), resulting in abnormally thick film (~88.9 µm) across the entire chip. (2) Optimized protocol with modified spin speeds and acceleration rate significantly suppressed edge-beading and eliminated rim-to-center flow, achieving near-target thickness (~14 µm). Despite these optimizations, the residual edge-beading remained high at ~19.4 µm (EBI = 162 %), necessitating a structural approach to further suppress edge-beading. We therefore designed an edge-beading suppression frame (EBSF) as an integrated solution. b) Photoresist thickness at three edge locations was measured before and after spin coating parameter modification using high-viscosity photoresist (without EBSF structure).


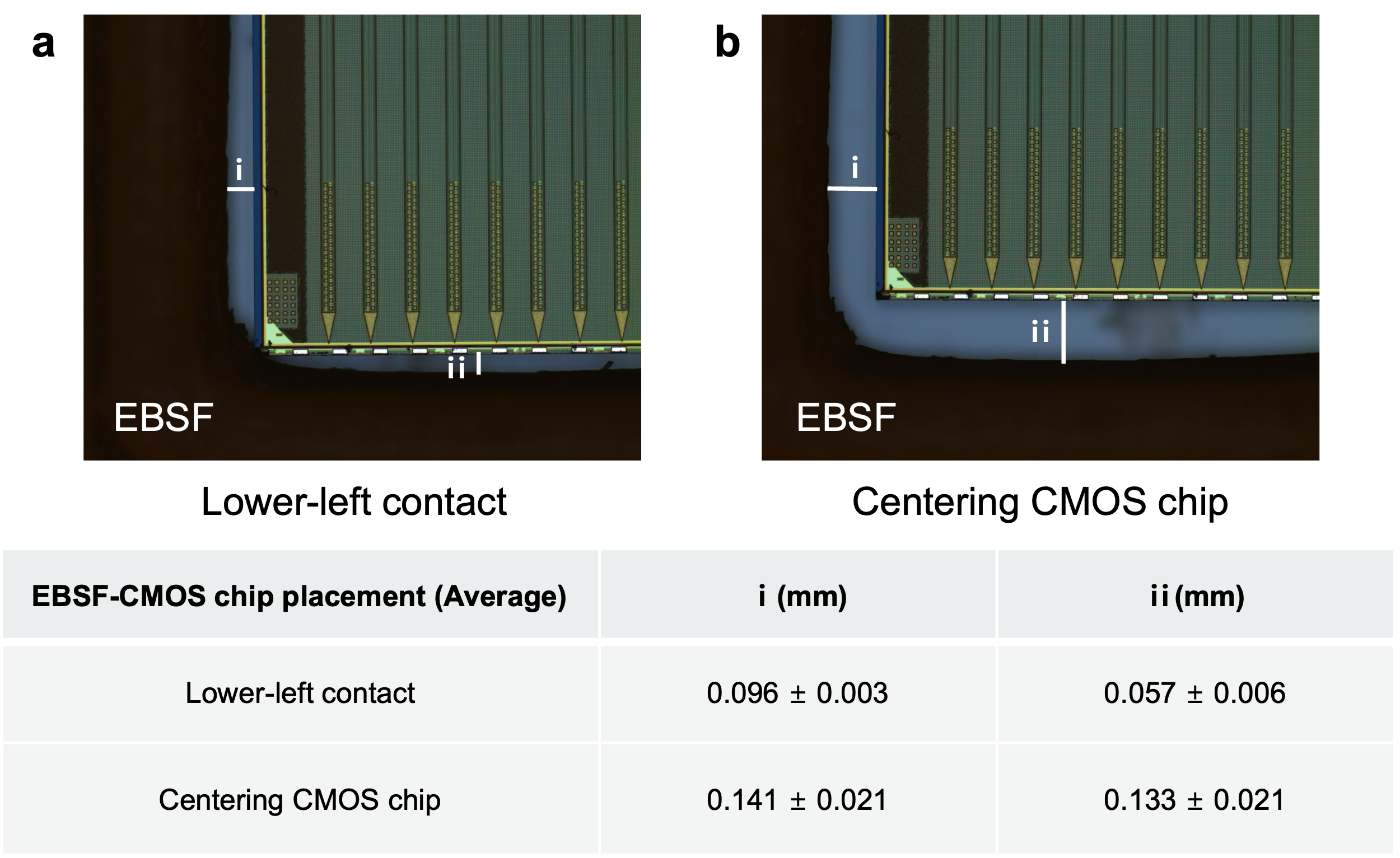


| **EBSF-CMOS chip placement (Average)** | **ⅰ(mm)** | **ⅱ(mm)** |
| --- | --- | --- |
| Lower-left contact | 0.096 ± 0.003 | 0.057 ± 0.006 |
| Centering CMOS chip | 0.141 ± 0.021 | 0.133 ± 0.021 |

**Figure S6 |** Edge-beading suppression frame (EBSF) placement optimization. To further mitigate edge-beading beyond spin coating parameter modification, we fabricated and implemented an edge-beading suppression frame (EBSF) that creates controlled gaps around the chiplet perimeter during photoresist deposition. Two placement configurations were evaluated to maximize edge-beading suppression efficiency: a) lower-left contact placement, where the chiplet was positioned against the frame's lower-left corner, creating two asymmetric gaps with measured margins of 0.096 ± 0.003 mm (left edge) and 0.057 ± 0.006 mm (bottom edge); and b) center contact placement, where the chiplet was centered within the frame, generating four symmetric gaps with margins of 0.141 ± 0.021 mm (horizontal) and 0.133 ± 0.021 mm (vertical) (n = 7-10 measurements per configuration). These controlled gap dimensions were designed to balance photoresist flow uniformity across the chip surface while spatially isolating edge-beading artifacts within the frame cavity, enabling subsequent quantitative comparison of edge-beading suppression performance between the two geometries.


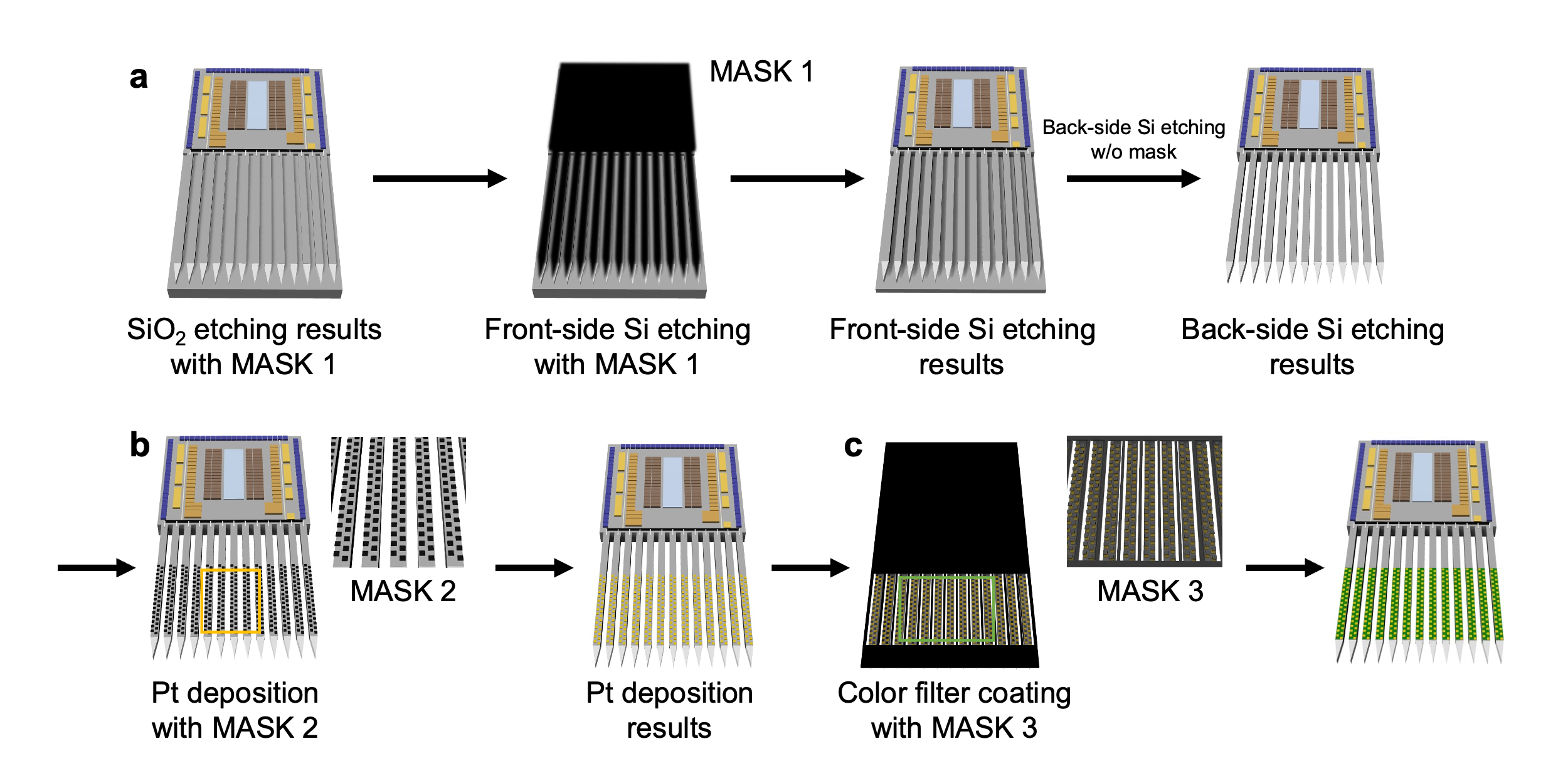


**Figure S7 |** Post-CMOS MEMS fabrication sequence: shank releasing, Pt electrode deposition, and color filter integration processes on the CMOS neural probe chiplet. The process employed three photolithography masks (MASK 1-3) with corresponding etching, deposition, and patterning steps. a) MASK 1 was used to pattern photoresist for protecting the shank regions while exposing inter-shank gaps. Dielectric etching removed the SiO_2_ BEOL stack between shanks with a target depth of 13.2 µm (12 µm + 10 % over-etching margin), verified by profilometry to confirm complete oxide clearance and Si substrate exposure. Using the same MASK 1 pattern, deep reactive ion etching (DRIE) of front- and back-side Si completed the release of the shanks from the chip body, yielding free-standing 2.4-mm-long shanks with 200 µm pitch. b) Passivation layers (Si_3_N_4_/SiO_2_) covering the Al electrode sites on each shank were selectively opened by reactive ion etching. MASK 2 photolithography protected all areas except the electrode openings (15 × 15 µm). E-beam evaporation deposited a Ti adhesion layer (15 nm) followed by Pt (100 nm) conformally over the patterned surface. Lift-off in acetone for an hour removed the photoresist and overlying Ti/Pt, leaving Pt deposits exclusively on the 32 electrode sites per shank, forming low-impedance neural recording interfaces. c) MASK 3 photolithography defined the photodiode array regions while keeping electrode sites exposed. A polymer-based color filter layer was spin-coated and patterned over the 64 paired photodiodes (8 × 8 µm, 19 µm pitch) on each shank, enabling wavelength-selective optical detection for multi-color imaging or optogenetic readout. The final device integrated Pt electrodes for electrophysiology and color-filtered photodiodes for optical sensing within a single monolithic CMOS platform.


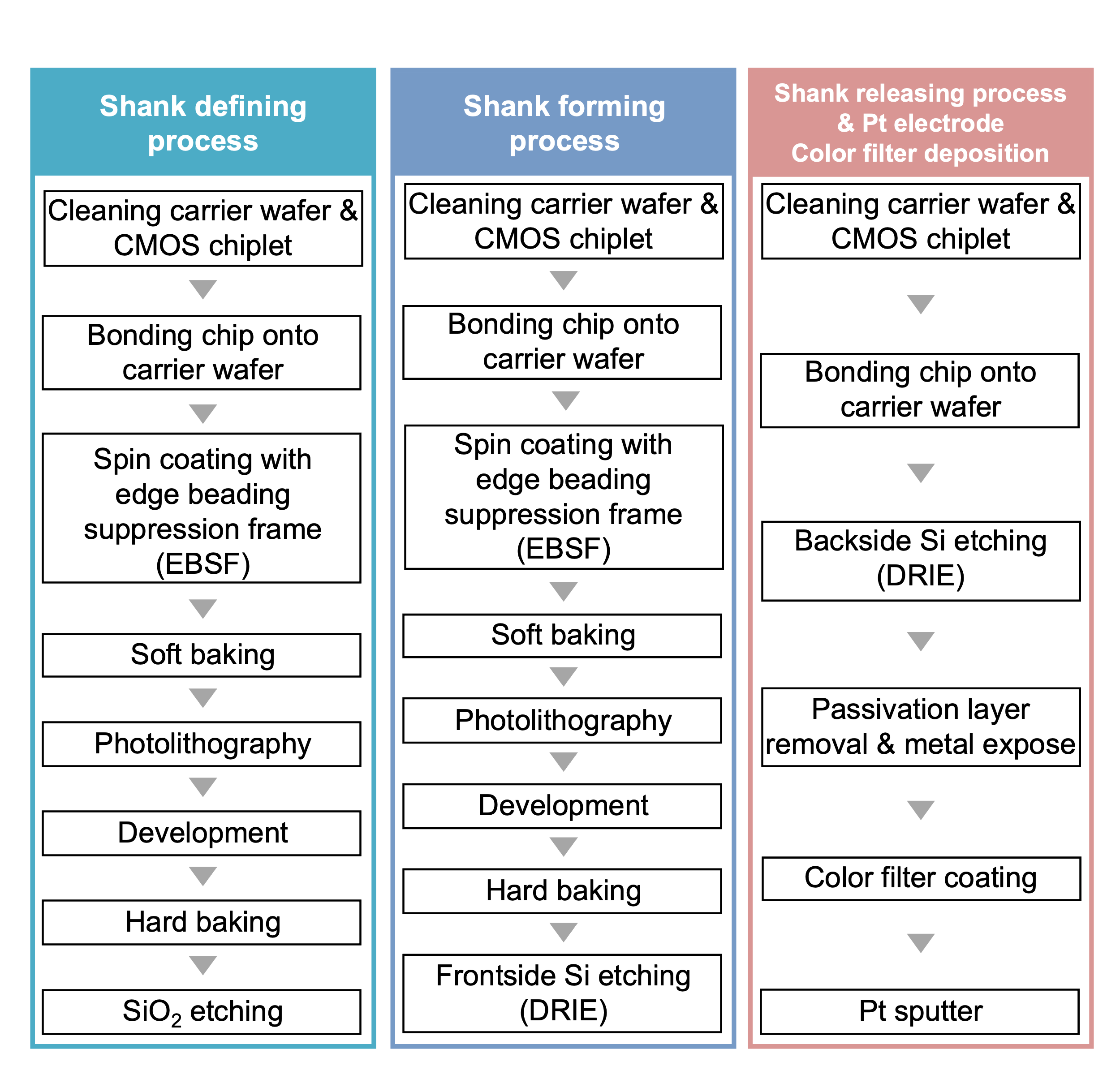
**Figure S8 |** Complete post-CMOS fabrication workflow transforming a monolithic CMOS chiplet into a multifunctional 13-shank neural probe. Beyond modifying spin-coating parameters and employing the EBSF, photolithography was further optimized via a ramped soft-bake of thick photoresist layers to prevent voids with 20-minute rehydration delays, implemented post-soft-bake and post-exposure. These intervals enable diffusion of photo-generated acidic species in DNQ/novolac resist, sharpening dissolution contrast and improving developer penetration while suppressing scumming in high-aspect-ratio features. Hard baking enhanced resist adhesion for dry etching. Shank defining, forming, and releasing processes: Dielectric etching process was conducted for shank defining, followed by Si etching with deep reactive ion etching (DRIE) process for shank forming and complete releasing processes. Pt electrodes deposition: The removal of passivation layer (Si_3_N_4_, SiO_2_) exposed 15 × 15 µm electrode sites. Ti (15 nm)/Pt (100 nm) e-beam evaporation and lift-off formed low-impedance contacts on 32 electrodes per shank. Color filters coating: Color filter with SU-8 photoresist was fabricated and patterned over 64 photodiodes (8 × 8 µm) per shank for wavelength-selective optical detection, creating a monolithic platform combining electrical recording and optical imaging capabilities.


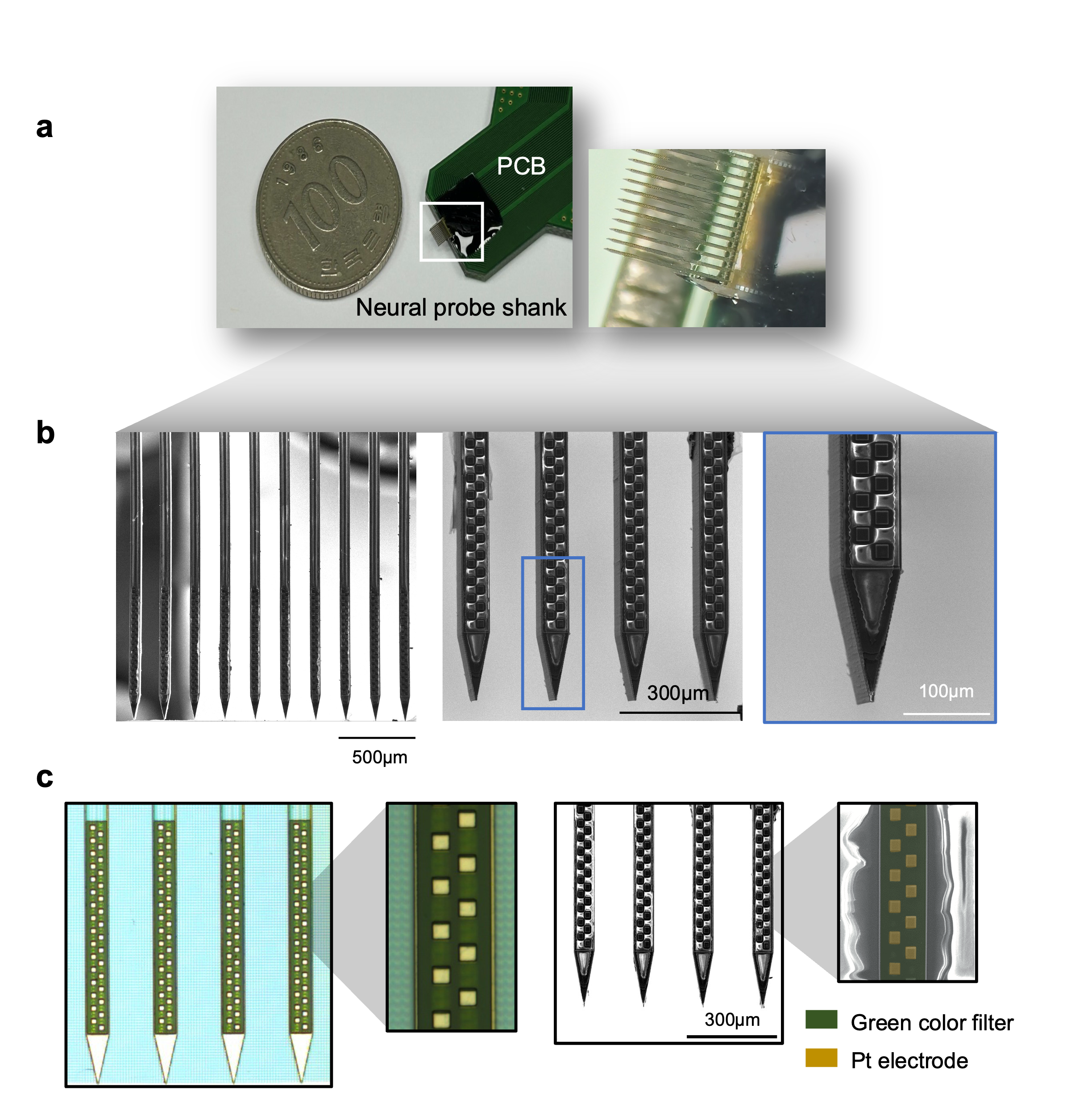


**Figure S9 |** Fabricated neural probe characterization: device integration, structural integrity, and functional component verification. Scanning electron microscopy (SEM) images of final fabricated neural probe. a) Images of the neural probe die wire-bonded onto a custom printed circuit board (PCB) carrier; bonding wires connect the on-chip pads to PCB traces. Neural probe was placed beside a coin as a scale reference, highlighting the ultra-high-density integration of 13-shank. b) High-magnification SEM images confirm that the shank geometry is precisely defined after processing without observable physical damage. c) Optical microscope (OM) image of the shank integrated with Pt electrode and color filter. SEM image with false-color for clarity–yellow color for Pt electrode and green color for color filter.


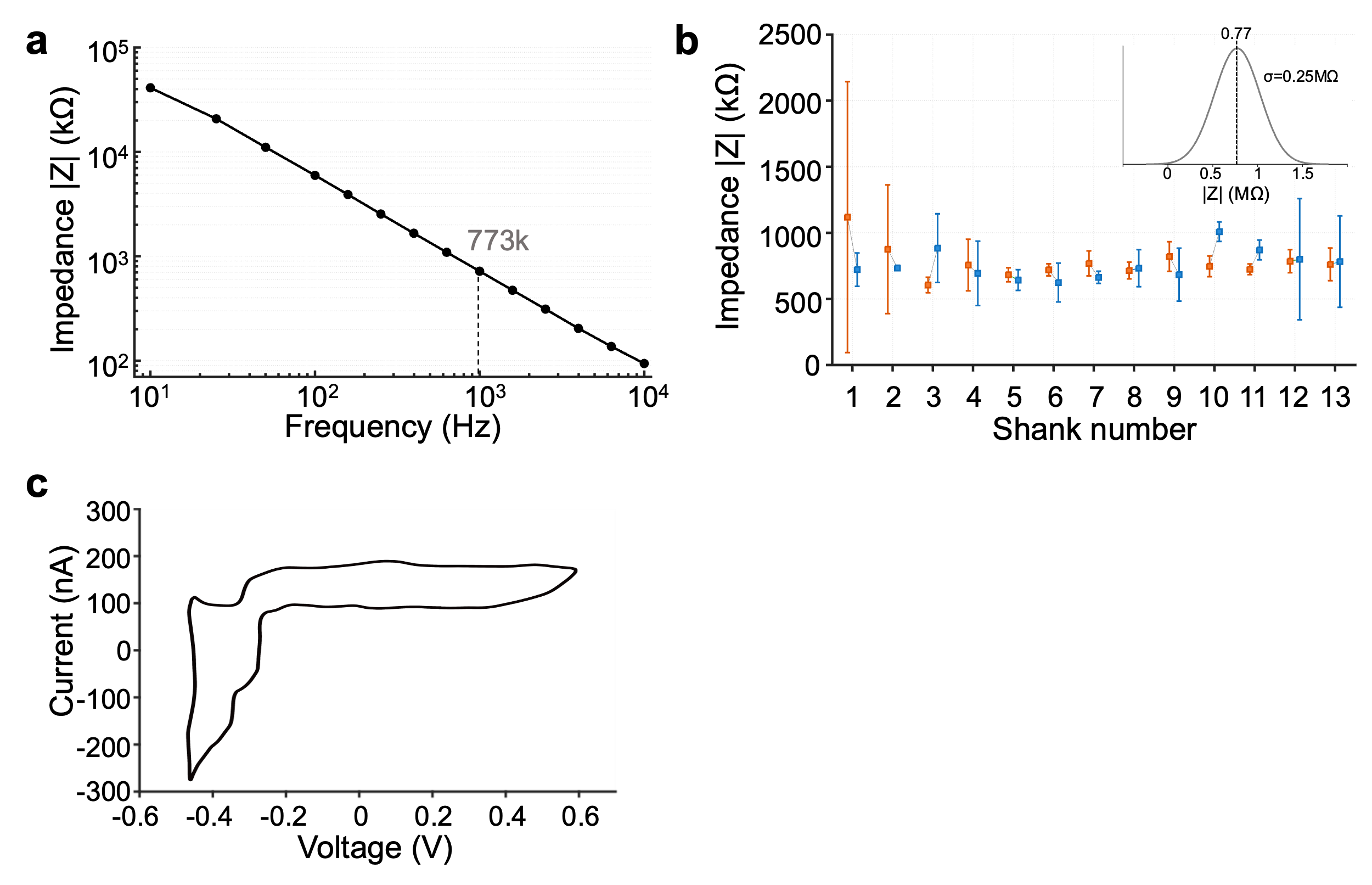


**Figure S10 |** a) Frequency-dependent impedance magnitude (|Z|) of electrodes across shanks measured in PBS (pH 7.4). (Autolab PGSTAT302N) b) Shank-wise impedance variation at 1 kHz across two chips. For each shank, the electrodes exhibiting the highest and lowest impedance values at 1 kHz were identified. The overall variation in impedance remains modest, demonstrating good fabrication consistency. Shanks located near the chip edge display relatively wider error bars, likely reflecting non-uniform electrode material deposition toward the wafer periphery. In addition, a small number of electrodes exhibit unusually low impedance values, which are most likely associated with measurement-setup effects, such as unintended parallel conduction during PBS-based impedance testing. c) Cyclic voltammetry (CV) curve of the fabricated metal electrode measured in PBS using a three-electrode configuration over the recording circuitry input range (–0.5 to 0.6 V). The response is predominantly capacitive within –0.2 to 0.6 V, exhibiting a quasi-rectangular profile without distinct redox peaks. A cathodic current increase appears below –0.2 V, indicating deviation from purely capacitive behavior. The normal recording range lies within the capacitive-dominant regime.


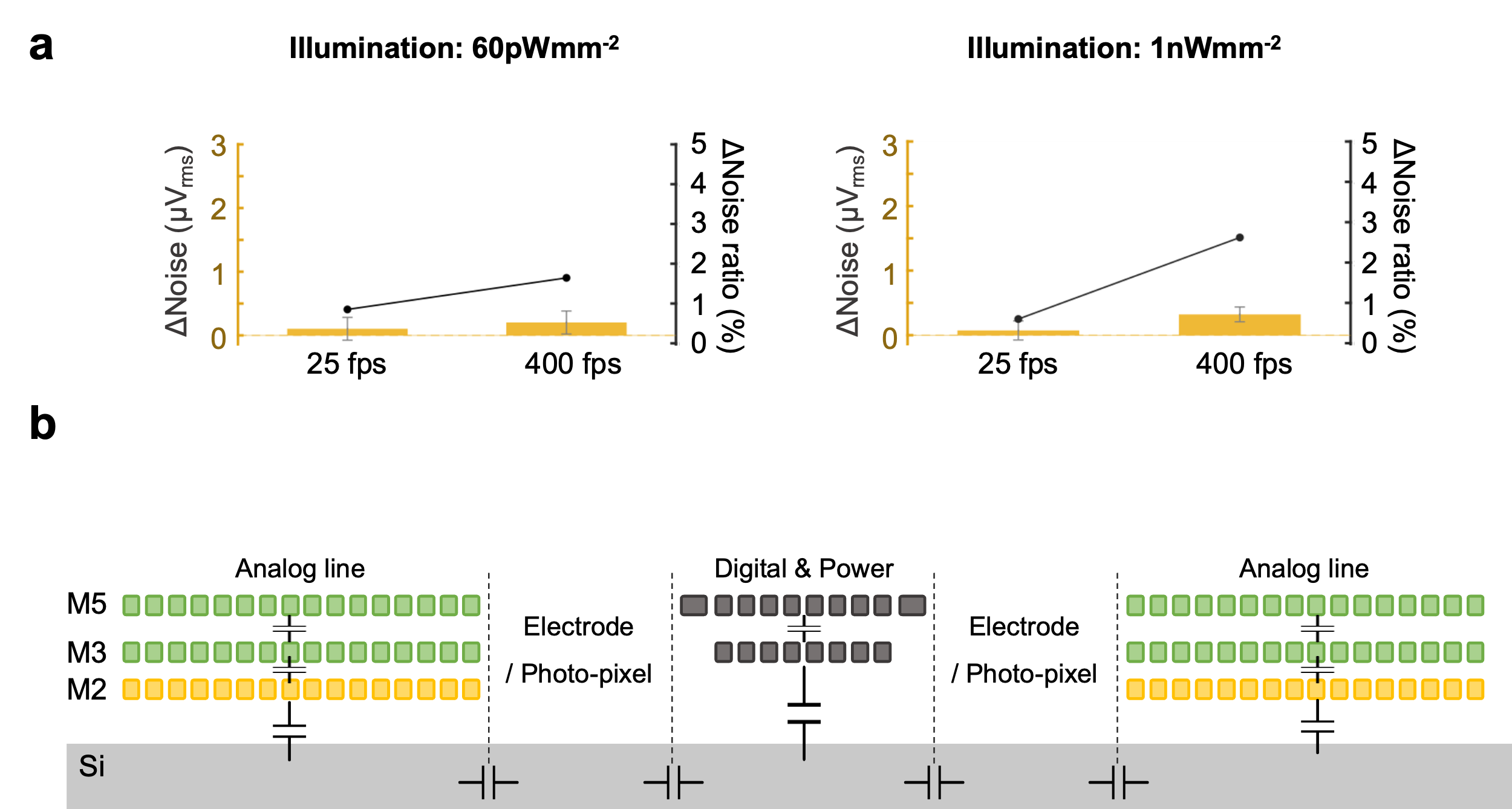


**Figure S11 |** a) Evaluation of photo-pixel-induced electrical interference under optical illumination. At 25 Hz, illumination at 60 pWmm^-2^ and 1 nWmm^-2^ resulted in RMS noise increases of 0.10 µV (0.8 %) and 0.07 µV (0.6 %), respectively, relative to baseline. At 400 Hz, increases of 0.20 µV (1.6 %) and 0.32 µV (2.6 %) were observed under the same conditions. Error bars represent standard deviation. b) Diagram illustrating capacitive coupling between neighboring signal lines in the shank layout.


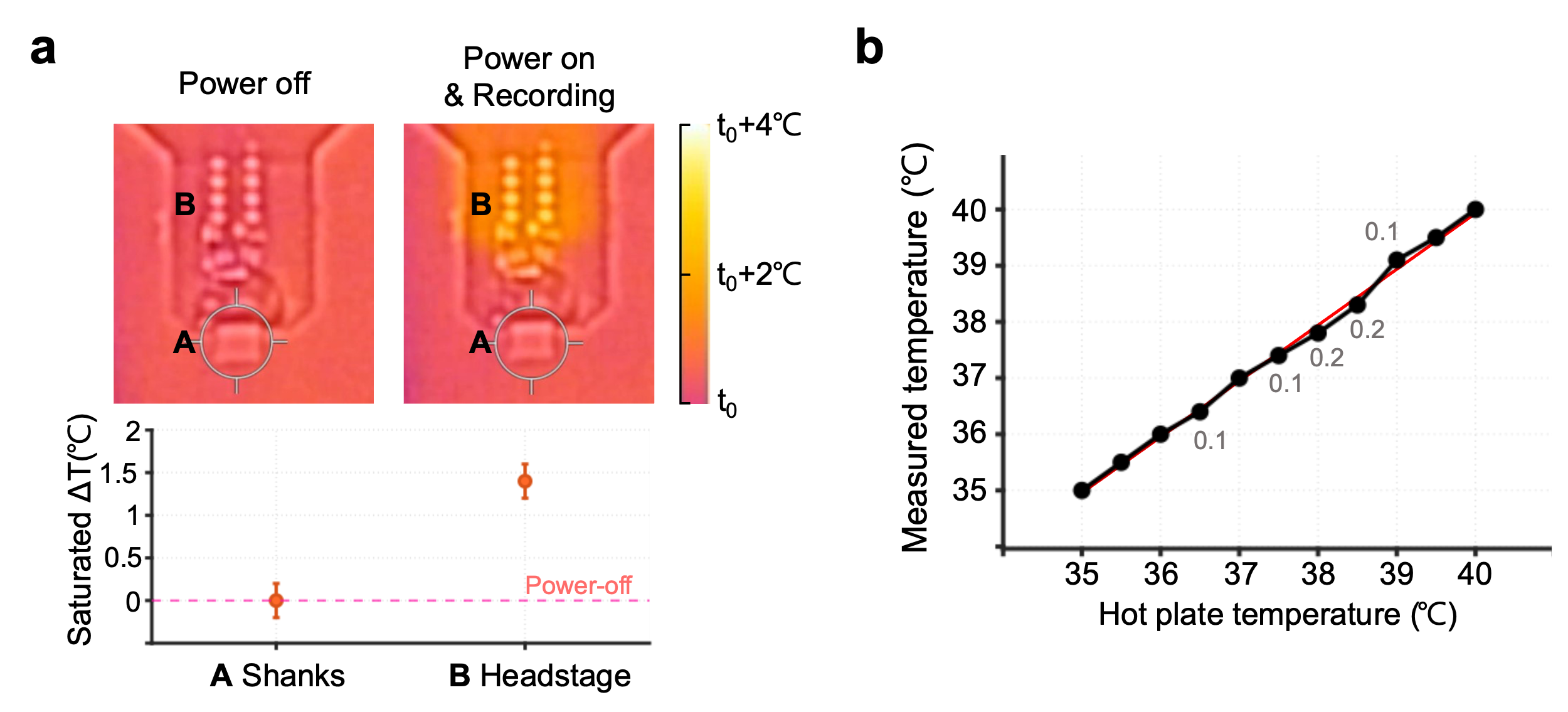


**Figure S12 |** Evaluation of device-induced temperature rise. a) Thermal imaging under power-off and active recording conditions. During simultaneous electrode and photodiode readout, the temperature increases at the implanted shank regions remained below 0.2 °C, while a localized rise (~1.4 °C) was confined to the non-implanted headstage. b) Validation of FLIR thermal imaging accuracy.


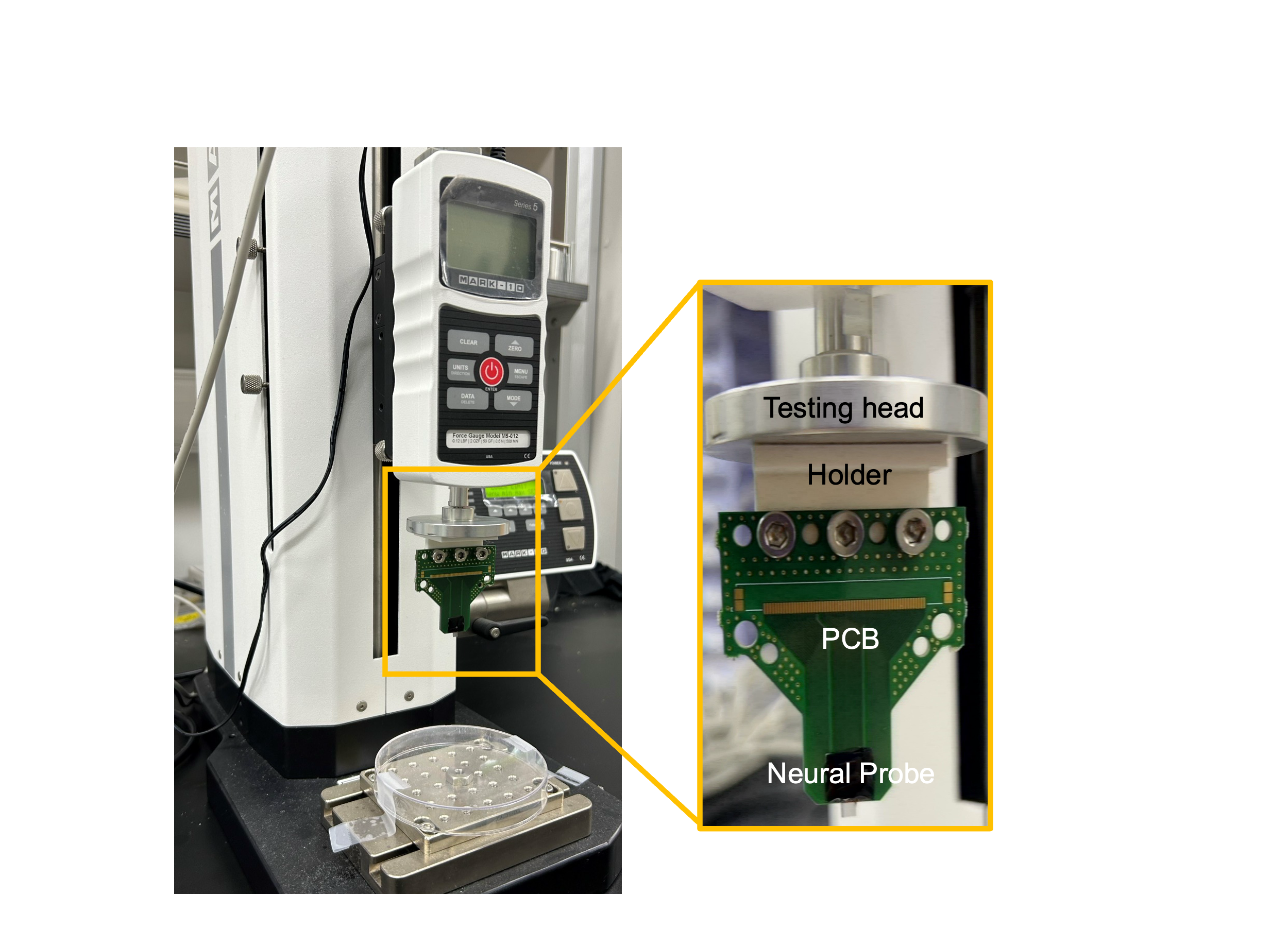
**Figure S13 |** Dura penetration force measurement setup for 13-shank multimodal CMOS neural probe. Penetration force for each shank was quantified using a digital force gauge (M5-012E, Mark-10, USA) mounted on a motorized test stand (ESM303E, Mark-10) while the wire-bonded neural probe was rigidly fixed to the actuator via a custom 3D-printed holder to eliminate fixture compliance. Mouse brain tissue with intact dura was positioned on the stand platen and aligned normal to the shank axis. Prior to testing, the load cell was zeroed under a no-contact condition, and the approach trajectory was calibrated to ensure perpendicular insertion. Insertion was performed at a constant approach rate of 1.1 mm/min, and penetration force was recorded continuously for each of the 13 shanks in sequence. This configuration enables reproducible assessment of dura breakthrough force and post-penetration force profiles while minimizing mechanical artifacts from probe mounting and stage motion.


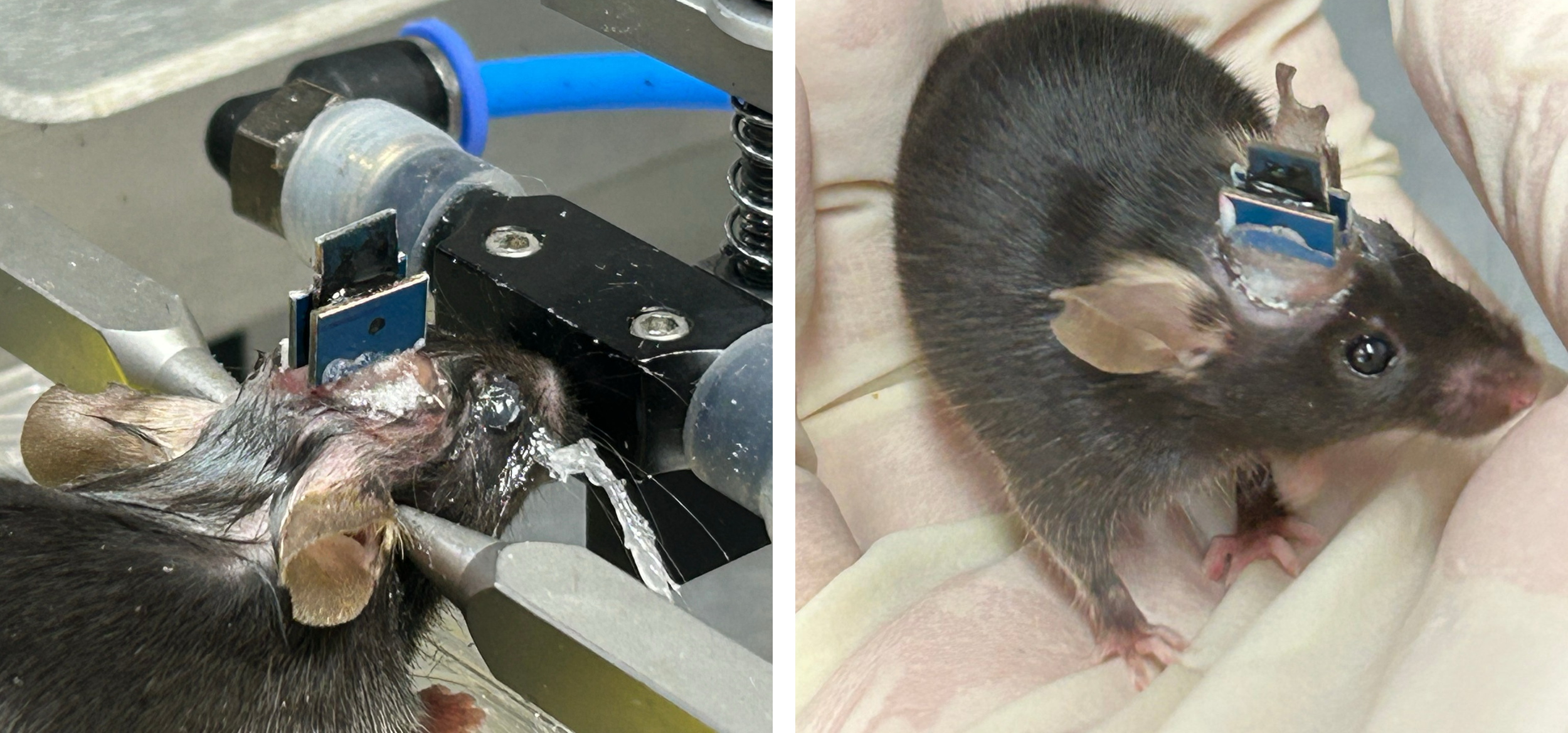


**Figure S14 |** For 1 week post-insertion immunohistochemical analysis and behavior test, the neural probe was secured in place using dental cement to ensure stable chronic implantation.


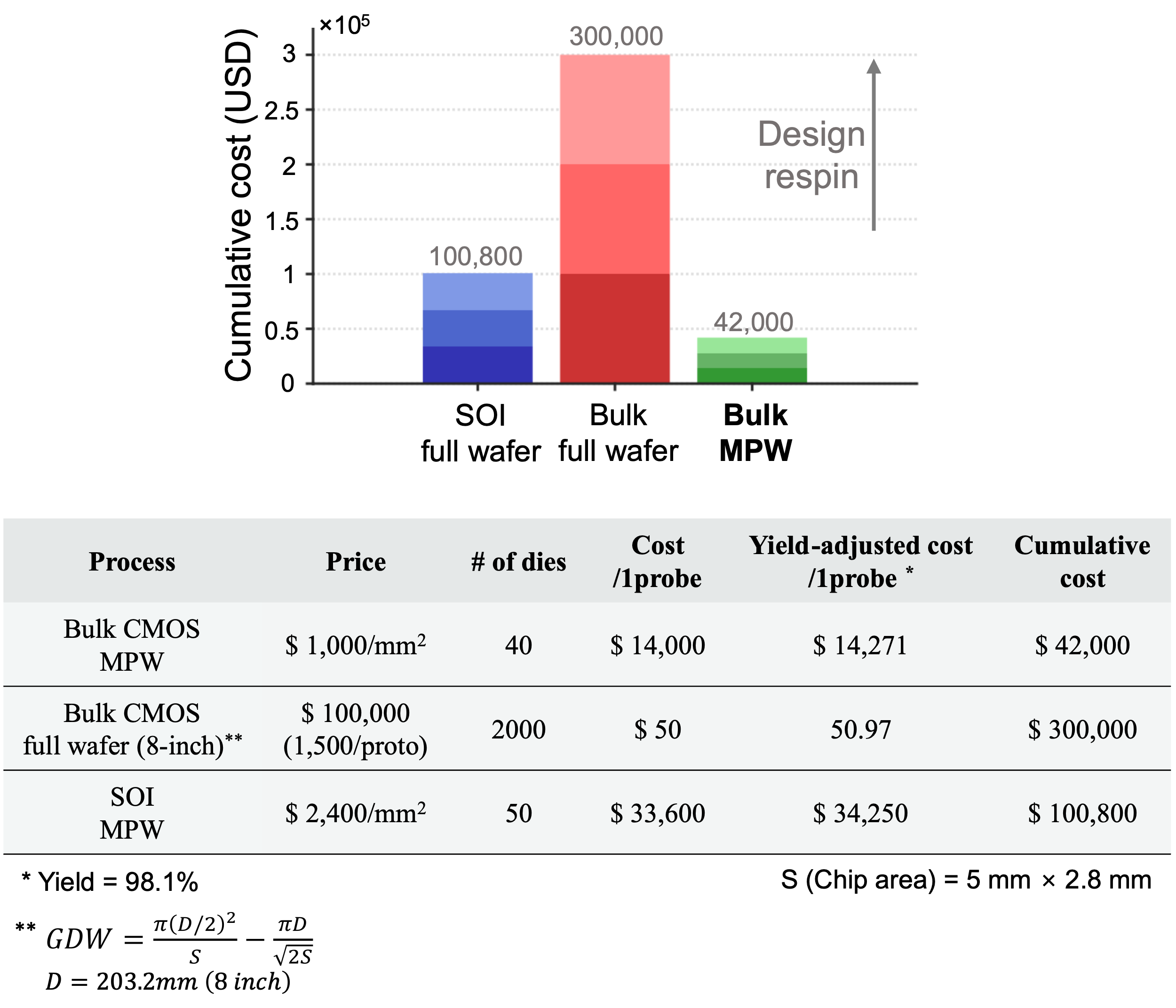


| **Process** | **Price** | **# of dies** | **Cost**  **/1probe** | **Yield-adjusted cost**  **/1probe^*^** | **Cumulative cost** |
| --- | --- | --- | --- | --- | --- |
| Bulk CMOS  MPW | $ 1,000/mm^2^ | 40 | $ 14,000 | $ 14,271 | $ 42,000 |
| Bulk CMOS  full wafer (8-inch)^**^ | $ 100,000  (1,500/proto) | 2000 | $ 50 | 50.97 | $ 300,000 |
| SOI  MPW | $ 2,400/mm^2^ | 50 | $ 33,600 | $ 34,250 | $ 100,800 |
| * Yield = 98.1%  ^**^ $GDW=\frac{\pi\left( D/2 \right)^{2}}{S}-\frac{\pi D}{\sqrt{2S}}$ $∵D=203.2mm \left( 8 inch \right)$ | | | | S (Chip area) = 5 mm × 2.8 mm | |

**Figure S15 |** The bar graph illustrates the cumulative fabrication cost assuming three independent design respins, comparing MPW and full-wafer production process. Pricing data for TSMC 180 nm bulk CMOS MPW and full-wafer (8-inch) runs were referenced from MUSE Semiconductor, while X-Fab 180 nm SOI MPW pricing was obtained from EUROPRACTICE. The table summarizes estimated cost per probe, yield-adjusted cost per probe, and total cumulative cost under each fabrication process. The yield is approximately 98.1 %, corresponding to a total loss probability of about 1.9 %. Notably, the probability of a color-filter deviation exceeding ± 10 % from the mean (4×10^-6^) is combined with the probability that the electrode impedance exceeds the 1.4 MΩ limit—defined to ensure less than −0.5 dB signal attenuation relative to the ~28 MΩ recording input impedance (1.9 %).

| **Table S1 \|** Cost summary table | | | |
| --- | --- | --- | --- |
| Category | Process | Cost | Notes |
| Post-processing | Shank defining | $ 27.18 | Cost of cleaning solution, developer, photoresist, and ABS dye are not included |
|  | Shank releasing/dicing | $ 72.47/41.08 |  |
|  | Electrode/filter formation | $ 34.15/24.39 |  |
| **Total Cost:** | | $ 199.27 |  |

**Table S1 |** Categorized and itemized the major cost components involved in our probe fabrication process—including CMOS fabrication and subsequent post-processing steps—to provide a transparent reference framework for readers to interpret the reported cost reduction and assess its broader applicability.

| **Table S2 \| Overall details of process** | | | | |
| --- | --- | --- | --- | --- |
| **Category** | **Process** | **Parameter** | **Used** | **Requirements** |
| **Foundry Backend-of-Line (BEOL)** | Metal stack | Top metal thickness | 4 µm | 1 µm ~ |
|  | Etch stopping layer | Material | Aluminum | (For fluorine-based plasma etching)  Al or Al alloys |
| **Post-CMOS Processing** | Chiplet and EBSF^*^ alignment | Gap between chiplet and frame | Horizontal: 0.096 ± 0.003 mm  Vertical: 0.057 ± 0.006 mm | Lower-left contact alignment |
|  | Spin coating | Target photoresist thickness | ~ 14 µm |  |
|  | Soft baking | Time | 60-80-100-80-60 ℃  1-1-5-1-1 min | Ramped baking |
|  | Reflow | Time | ~ 20 min |  |
|  | Exposure | Power | SiO_2_ etching: 1700 mJ/cm^2^  Si etching: 2720 mJ/cm^2^ |  |
|  | Reflow | Time | 20 min ~ |  |
|  | Development | Time | 4 min |  |
|  | Hard baking | Temperature and time | 100 ℃  3 min |  |
|  | Front-dielectric etching | Target material & depth | 12 µm + 10% overetching |  |
|  | Front-Si etching | Target depth | 180 µm |  |
|  | Back-Si etching | Target depth | 280 µm |  |
| * EBSF: edge-beading suppression frame | | | | |

**Table S2 |** Consolidated process parameters for foundry and post-CMOS processing.

| **Table S3 \|** Details for Si, SiO_2_ DRIE process | | | |
| --- | --- | --- | --- |
| **Process** | **Etching target** | **Gas** | **Etch rate** |
| Dielectric layer (SiO_2_) etching | 13.2 µm (12 µm + 10 % over etching) | CHF_3_ 90 sccm, Ar 10 sccm | 53 Å / sec  300 s 8 cycles |
| Frontside deep trench Si reactive ion etching (DRIE) | 180 µm | C_4_F_8_, SF_6_, O_2_ | 0.5 µm / cycle  1 hr 30 min |
| Backside deep trench Si reactive ion etching (DRIE) | 280 µm |  |  |

**Table S3 |** Details for Si, SiO_2_ etching process. This table summarizes etching sequence, target etching thickness, and key parameters required for each process step–gas chemistry/flow, chamber pressure, and etch rate.

| **Table S4 \|** Spin coating details for Si, SiO_2_ etching profile | | | | |
| --- | --- | --- | --- | --- |
| Purpose | Step | Photoresist type | Speed (rpm) | Time (sec) |
| Photoresist bonding with wafer and chip | 1 | AZ GXR-601  (46 cP) | 500 | 5 |
|  | 2 |  | 500 | 5 |
|  | 3 |  | 3000 | 5 |
|  | 4 |  | 3000 | 30 |
| Photoresist mask for SiO_2_, Si etching | 1 | AZ 10XT 520 cP | 1000 | 2 |
|  | 2 |  | 1000 | 30 |
|  | 3 |  | 5000 | 2 |
|  | 4 |  | 5000 | 5 |

**Table S4 |** Details required for spin coating process–photoresist type, first, second spin speed and acceleration profile.

| **Table S5 \|** Photolithography details for Si, SiO_2_ mask | | | |
| --- | --- | --- | --- |
| Process | Soft baking (min) | **Exposure dosage (mJ/**$\mathbf{cm}^{\boldsymbol{2}}$**)** | Development (min) |
|  | **60 ℃ 80 ℃ 100 ℃ 80 ℃ 60 ℃** |  |  |
| Mask for SiO_2_ etching | 1 1 5 1 1 | 1700 | 4 |
| Mask for Si etching | 1 1 5 1 1 | 2720 | 4 |

**Table S5 |** Procedure details for soft baking and photolithography, followed by development.

| **Table S6 \|** Details for SiO_2_, Si_3_N_4_ RIE process | | |
| --- | --- | --- |
| Process | Etching target | **Gas** |
| Passivation layer (SiO_2_, Si_3_N_4_) reactive ion etching (RIE) | Si_3_N_4_ 0.6 µm  SiO_2_ 3.75 µm | CHF_3_, CF_4_, O_2_ |

**Table S6 |** Passivation layer removal details to expose the electrodes place on the shank–etching layer materials, etching target, and required gas for reactive ion etching (RIE).

| **Table S7 \|** Details for Pt E-beam evaporation | | | | | | |
| --- | --- | --- | --- | --- | --- | --- |
| Process | Photoresist type | Spin coating (rpm) | Soft baking (min) | **Exposure dosage (mJ/**$\mathbf{cm}^{\mathbf{2}}$**)** | PEB (min) | Development (min) |
|  |  |  | **90℃** |  | **110℃** |  |
| Ti/Pt E-beam evaporation | DNR-L300-40 (120 cP) | 1000/4000 | 1.5 | 182 | 1.5 | 2 |

**Table S7 |** Process details for Pt E-beam evaporation. For the patterning preparation before performing E-beam evaporation, parameters of spin coating, soft baking, photolithography, post-exposure bake (PEB), and development were mentioned in this table.

| **Table S8 \|** Process details for SU-8 green color filter | | | | | | |
| --- | --- | --- | --- | --- | --- | --- |
| Process | Thickness (µm) | Spin coating (rpm) | Soft baking (min) | **Exposure dosage (mJ/**$\mathbf{cm}^{\boldsymbol{2}}$**)** | PEB (min) | Development (min) |
|  |  |  | **65 ℃ 95 ℃** |  | **65 ℃ 95 ℃** |  |
| SU-8 2002 green color filter | 2.54 | 500/3000 | 5 10 | 2640 | 5 10 | 1.5 |

**Table S8 |** Details of color filter deposition process. With standard MEMS process (e.g. spin coating, photolithography), color filter array was coated on the photodiode with the thickness of 2.54 µm.

| **Table S9 \|** Neural probe comparison table | | | | | | | |
| --- | --- | --- | --- | --- | --- | --- | --- |
|  | | | Chou et. al.,  2022 | Jun et. al.,  2017 | Yilmaz et. al.,  2025 | Angotzi et. al.,  2025 | This work |
| Process/technology | | | Research  Fab | SOI CMOS  130nm | Bulk CMOS  130nm | Bulk CMOS  180nm | Bulk CMOS  180nm |
| Geometry | Number of shanks | | 3 | 1 | 2 | 4 / 8 | 13 |
|  | Shank Dimensions  W×L (mm) | | 1×6.55 | 0.07×10 | 0.4×4.1 | 2.2×5 | 2.6×2.4 |
| Optical | Number of pixels | | 2 | - | 512 | - | 832 |
|  | Pixel size | | 60 × 172 μm^2^ |  | 46.5 μm^2^  (d = 7.7 μm) |  | 8 × 8 μm^2^ |
|  | Simultaneous rec. | | 2 (off-chip) |  | 512 |  | 64 |
|  | SNR  condition specified | | 7.6 |  | 5.5 @ V_ex_=1V  1nWmm^-2^ |  | 62.5  @ 1nWmm^-2^ |
|  | Min detectable irradiance  (nWmm^-2^) | | 2 |  | 0.033 |  | 0.001 |
|  | Saturation irradiance  (nWmm^-2^) | | 150 |  | 2.1 |  | 25 |
|  | Dynamic range (dB) | | 18.75 |  | 18.04 |  | 43.98 |
| Electrical | Number of recording site | | 21 | 960 | - | 1024 | 416 |
|  | Simultaneous rec. | | 16 (off-chip) | 384 |  | 1024 | 32 |
|  | Input-referred noise | LFP | - | 10.3 μV  @ 0.5-1k |  | 16.5±3.8 μV  @ 0.1-300 | 5.83±2.1 μV  @ 1-300 |
|  |  | AP |  | 6.4 μV  @ 300-10k |  | 6.7±1 μV  @ 300-5k | 10.8±0.7 μV  @ 300-3.7k |
|  | Electrode size | | 20 × 20 μm^2^ | 12 ×12 μm^2^ |  | 14 ×14 μm^2^ | 17 ×17 μm^2^ |
| Power consumption | | | - | 15 mW | 6.24 mW | 6.14 mW | 8.24 mW |

**Table S9 |** Comparative analysis of various neural probe architecture.

**References**

[1] X. Man, N. Bao, Y. Hao, Y. Feng, X. Ma, “Study of SiO_2_ Etching Processing with CH_4_ /SF_6_ Plasmas” *Physica Status Solidi* (2020) *217*: 2000223.

[2] I. Nesterenko, B. Kalas, T. D. Dao, J. Schulze, N. Andrianov, “Mechanism of selective SiO2/photoresist reactive ion etching in an inductively coupled plasma operated in a C4F8/H2 gas mixture” *Applied Physics Letters* *126* (2025): 031603.

[3] N. Atthi, O. Nimittrakoolchai, W. Jeamsaksiri, S. Supothina, C. Hruanun, A. Poyai, “Study of optimization condition for spin coating of the photoresist film on rectangular substrate by Taguchi design of an experiment” *Songklanakarin Journal of Science and Technology* 31 (2009): *331-335*.

[4] C. A. Mack, “Resolution and depth of focus in optical lithography” *Microlithographic Techniques in IC Fabrication* (SPIE, 1997), 14-27.

[5] Q. Liu, P. Zhou, “Effect of recessed chuck slits on the edge buildup in spin coating for rectangular substrates” *Progress in Organic Coatings 200 (*2025): 108994.

[6] C. J. Lawrence, W. Zhou, “Spin coating of non-Newtonian fluids” *Journal of Non-Newtonian Fluid Mechanics* 39 (1991): 137-187.

[7] L. E. Stillwagon, R. G. Larson, “Planarization during spin coating” *Physics of Fluids A: Fluid Dynamics* 4 (1992): 895-903.

[8] D. E. Bornside, C. W. Macosko, L. E. Scriven, “Spin coating: One-dimensional model” *Journal of Applied Physics* 66 (1989): 5185-5193.

[9] N. P. Pham, E. Boellaard, J. N. Burghartz, P. M. Sarro, “Photoresist coating methods for the integration of novel 3-D RF microstructures” *Journal of Microelectromechanical Systems 13* (2004): 491-499.

[10] O. Nalamasu, E. Reichmanis, J. Hanson, R. Kanga, L. Heimbrook, A. Emerson, F. Baiocchi, S. Vaidya, “Effect of post‐exposure delay in positive acting chemically amplified resists: An analytical study” *Polymer Engineering & Science* 32 (1992): 1565-1570.

[11] R. Dammel, *Diazonaphthoquinone-based Resists* (SPIE Press 1993).
